# When Just One Phosphate Is One Too Many: The Multifaceted Interplay between Myc and Kinases

**DOI:** 10.3390/ijms24054746

**Published:** 2023-03-01

**Authors:** Dalila Boi, Elisabetta Rubini, Sara Breccia, Giulia Guarguaglini, Alessandro Paiardini

**Affiliations:** 1 Department of Biochemical Sciences, Sapienza University of Rome, 00185 Rome, Italy; 2 Institute of Molecular Biology and Pathology, National Research Council of Italy, Sapienza University of Rome, 00185 Rome, Italy

**Keywords:** Myc, kinases, PLK1, Aurora-A, Aurora-B, GSK-3, PKA, PIM, BRD4, kinase inhibitors

## Abstract

Myc transcription factors are key regulators of many cellular processes, with Myc target genes crucially implicated in the management of cell proliferation and stem pluripotency, energy metabolism, protein synthesis, angiogenesis, DNA damage response, and apoptosis. Given the wide involvement of Myc in cellular dynamics, it is not surprising that its overexpression is frequently associated with cancer. Noteworthy, in cancer cells where high Myc levels are maintained, the overexpression of Myc-associated kinases is often observed and required to foster tumour cells’ proliferation. A mutual interplay exists between Myc and kinases: the latter, which are Myc transcriptional targets, phosphorylate Myc, allowing its transcriptional activity, highlighting a clear regulatory loop. At the protein level, Myc activity and turnover is also tightly regulated by kinases, with a finely tuned balance between translation and rapid protein degradation. In this perspective, we focus on the cross-regulation of Myc and its associated protein kinases underlying similar and redundant mechanisms of regulation at different levels, from transcriptional to post-translational events. Furthermore, a review of the indirect effects of known kinase inhibitors on Myc provides an opportunity to identify alternative and combined therapeutic approaches for cancer treatment.

## 1. Introduction

c-Myc and the other protein family members (i.e., N-Myc and L-Myc), collectively known as “Myc”, are ubiquitous basic helix–loop–helix–leucine zipper (bHLH-LZ) transcription factors that are critical for several cellular processes during cancer genesis and progression [1]. Indeed, Myc plays a central role among the molecular factors that drive tumour progression. c-Myc was first described as a viral oncoprotein able to induce myelocytomatosis in chickens after retroviral infection, and soon its derivation from a highly conserved vertebrate cellular gene was demonstrated [2,3]. The other members of the Myc family were subsequently discovered: N-Myc in neuroblastoma [4,5] and L-Myc in small cell lung carcinoma [6]. Since then, the Myc family members have been widely recognized as potential oncogenes and have been thoroughly studied for their structure, function, and regulation (as described in more detail by others [7,8]). Decades after its discovery, Myc continues to impress with its involvement in diverse pathways. The complexity of the Myc protein interaction network is reflected in the great heterogeneity of their transcriptional program and biomolecular activities [7,8,9].

**Figure 1:**
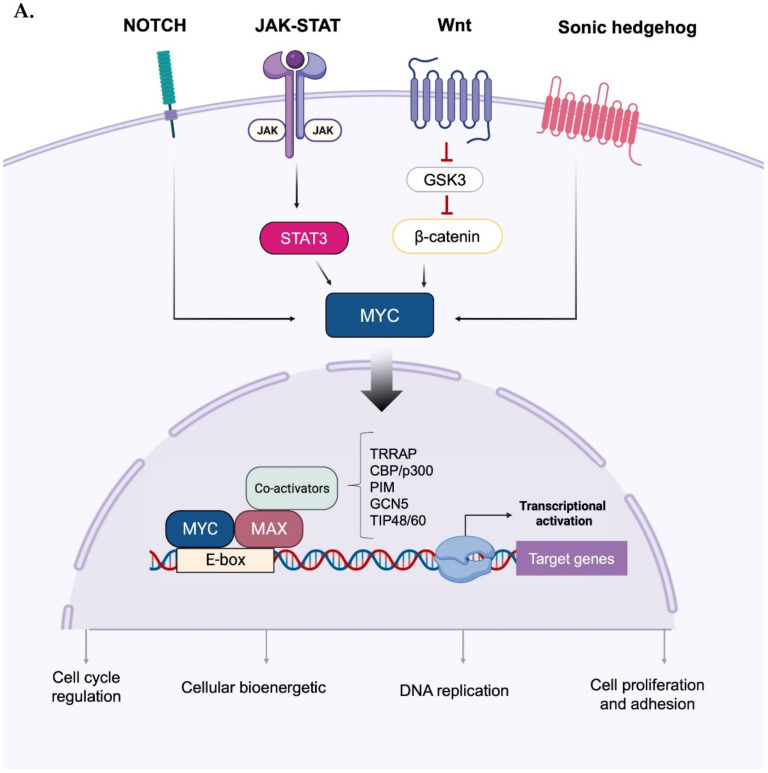

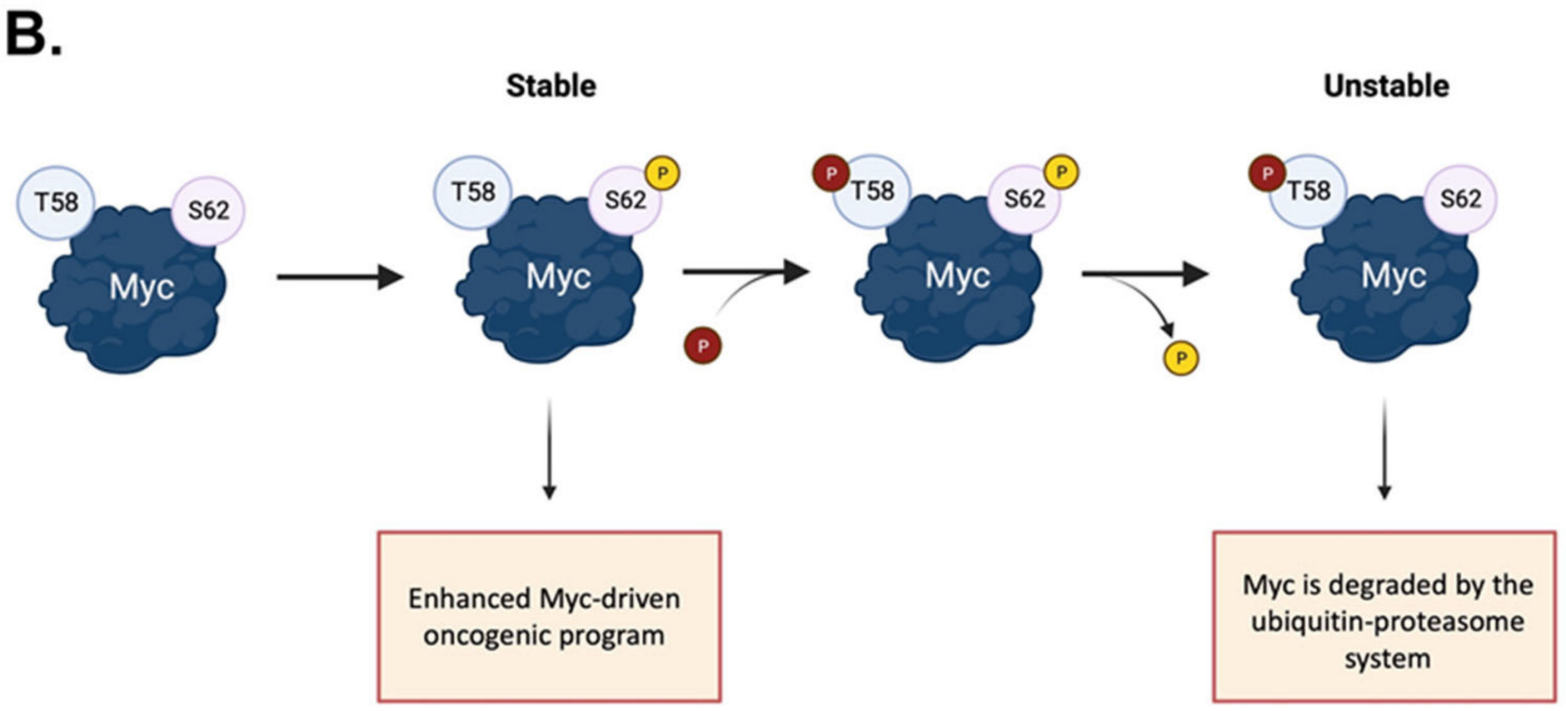
Activation and phospho-dependent stabilization of Myc. (**A**) Myc can be activated in response to different stimuli via several transduction pathways converging to Myc stabilization. Stabilized Myc associates with its protein partner MAX to form a heterodimer, which, together with other co-activators, binds to the E-box elements and drives the transcription of a target gene subset involved in a wide range of cellular processes. (**B**) Phospho-dependent stabilization of Myc. Myc displays two key phosphorylation sites that undergo hierarchical phosphorylation, supervising protein stability. Phosphorylation of Myc at the S62 residue determines protein stabilization and primes the subsequent phosphorylation at T58, which induces the removal of the phosphate group at S62; the unstable, singly phosphorylated T58-Myc is then recognized by the ubiquitin ligase Fbxw7 and degraded by the ubiquitin–proteasome system. Created with BioRender.com (accessed on 20 January 2023).

Upon forming an obligate heterodimer with its protein partner Max, Myc binds to the consensus DNA sequence 5′-CACGTG-3′ (known as the E-box) and drives the expression of its target genes [10] (Figure 1A). As a mild transcriptional modulator, the effects of Myc are amplified through interactions with a wide range of cofactors, co-activators, and chromatin remodelling components [11]. Relevant functional categories of MYC-induced genes include cell cycle control, DNA replication, cell growth and adhesion, cellular bioenergetics (e.g., glycolysis and mitochondrial biogenesis), and anabolic metabolism (e.g., synthesis of amino acids, nucleotides, and lipids) [1,12,13]. In addition, Myc and HIF1 actively induce the expression of glucose transporters and glycolytic enzymes [14], and the former acts as a downstream effector of several signalling cascades: for instance, aberrant Wnt/β-catenin signalling leads to the constitutive, high transcription of the MYC gene [15,16]; similarly, potent induction of MYC transcription occurs in response to perturbations of the Sonic hedgehog [17], Notch [18,19], and Janus kinase (JAK)— signal transducer and activator of transcription 3 (STAT3) pathways [20,21].

The pleiotropic activities of Myc make its tight regulation mandatory in healthy cells, which need to rapidly turnover Myc proteins. However, steady Myc levels may be affected by pathways that are disrupted in cancer, leading to its enhanced expression and increased stability [22]. Myc mRNA levels are regulated by both transcription initiation (which starts from different promoters [23]) and elongation [24], yielding a transcript that is highly unstable, with a half-life of 20–30 min [25]. Additionally, Myc is rapidly degraded after its synthesis, with a turnover of 25 min [26]. Posttranslational modifications, i.e., acetylation and methylation, contribute to regulating Myc activity and stability [27]. SUMOylation, consisting of the addition of ubiquitin-like molecules to the target protein, is another intriguing observed modification in Myc. The role of the SUMOylation in regulating Myc functions was debated; Myc is SUMOylated in more than 10 lysine residues [28,29,30], but the mutation of all these residues did not abolish Myc SUMOylation, nor alter its activity [28,29,30]. In a particular case, an increased Myc transcription activity was observed: in B cell lymphoma, the SUMOylation mediated by the E3 ligase PIAS1 allows the recognition and phosphorylation of Myc in S62 by JNK1, which precludes the oncoprotein degradation [31]. Noteworthy, in this tumour, a Myc-dependent transcriptional activation of SUMO cascade enzymes was observed. The overall SUMOylation induces G2/M arrest and consequent mitotic abnormalities, which are attributed to the Aurora kinases’ SUMOylation [32]. SUMOylation could also have a role in Myc protein stability, since the inhibition of the proteasome has been shown to increase the levels of SUMOylated Myc [28,33]. Moreover, the SUMO-specific protease SENP1 has been described to de-SUMOylate Myc, stabilizing the protein [33]. It is possible that the de-SUMOylation drives the subsequent de-ubiquitination on the same residues, since SUMO and ubiquitin modifications can exist on the same lysine residues [34].

The phosphorylation and dephosphorylation of key residues provide the main dynamic regulation of Myc cellular functions: its phosphorylation plays a key role in promoting Myc binding to DNA and in inducing its degradation. The conserved region of Myc called the Myc Box (MB) I is mainly involved in its ubiquitination and degradation [35] (Figure 1B). It contains the residues S62 and T58, which undergo cascade phosphorylation, regulating protein stability and functionality. S62 is a target for a variety of kinases, including ERK, CDKs, and JNK [36], and its phosphorylation correlates with the recruitment of Myc to specific promoters in response to oxidative stress. The S62A mutation has been shown to significantly alter an array of genes involved in apoptosis, proliferation, cellular bioenergetic, and signalling pathways [37]. The phosphorylation of S62 recruits the GSK-3β kinase, which, in turn, phosphorylates T58 on Myc, ensuring its recognition by the Fbxw7 ubiquitin ligase for degradation [38]. T58 and S62 are hotspots of mutation in lymphomas [39,40] and solid tumours [41,42]. The non-phosphorylatable T58A mutation leads to the accumulation of pS62 Myc, which presumably repulses Fbxw7 and increases Myc half-life to up to 120 min [40]. Additionally, Myc phosphorylated on T58 reduces the expression of BIM, which is particularly relevant as a tumour suppressor in MYC-driven B cell leukaemia [43]. The phosphorylation of other highly conserved sites, T358, S373, and T400, by the p21-activated kinase PAK2 has been reported to negatively impact on the Myc transcriptional program by interfering with the formation of the Myc–Max–DNA ternary complex [44,45].

In addition to MBI, other conserved motifs control Myc functions. MBII allows interaction with partners required for transcriptional regulation [46], while MBIII is important for cellular transformation [47]. Finally, MBIV overlaps with the nuclear localization signal [48]. Each motif is conserved among species and ensures the binding of Myc proteins with different partners. Interestingly, the MBI and the MBII motifs overlap with the transactivation domain (TAD), highlighting a well-established correlation between Myc protein stability and transcriptional activity [49].

The importance of kinases in Myc regulation goes beyond their ability to phosphorylate the protein. Some kinases can also indirectly affect Myc protein stability by inducing the degradation of the ubiquitin ligase (PLK1 and PKA, [50,51]). Additionally, some kinases physically interact with Myc, protecting it from proteasomal degradation, such as Aurora-A in neuroblastoma [52]. The cell-cycle-dependent Aurora-A and Aurora-B form a circuit with Myc, in which Aurora primes the oncogenic program of Myc and vice versa [53,54]. Aurora-A is also involved in Myc modulation in PKA-dependent tumours [55]. Other kinases are crucial for Myc transcriptional activity (i.e., PIM and BRD4 [56,57,58]). At the same time, Myc enhances the expression of protein kinases, creating a positive feedback loop.

In this review, we will delve into the cross-regulation between Myc and kinases, aiming at providing a comprehensive understanding of the complex signalling events between oncogenic proteins and their partners. We will identify common mechanisms of action and highlight altered oncogenic pathways. We will also describe the effects of some kinase inhibitors on Myc and explore the potential benefits of combining therapies to improve the selectivity of approaches to eradicate Myc-driven cancers.

## 2. Direct and Indirect Myc Regulation by Mitotic Kinases

The overexpression or deregulation of mitotic kinases is often associated with cancer progression. Defective cell cycle regulation can lead to uncontrolled cell division and chromosomal instability, making tumours hyper-proliferative and resistant to growth inhibition. As they are druggable, targeting mitotic kinases has been considered a promising strategy for anticancer intervention [59,60]. In this perspective, we describe the unusual involvement of classical mitotic kinases in promoting the survival of Myc-driven cancers.

### 2.1. PLK1 Kinase

PLK1 is a member of the Polo-like kinases (PLKs) family, which consists of five paralogues: PLK1, PLK2, PLK3, PLK4, and PLK5. It is a conserved serine/threonine kinase that is critical for the proper execution of mitotic events and the maintenance of genome stability during cell division [61,62]. The overexpression of PLK1 is a hallmark of many types of human cancers, including melanoma, ovarian carcinoma, breast, prostate, thyroid cancers, and glioma [63,64,65], and it is associated with chemoresistance and poor patient outcomes. Genetic ablation or inhibition of PLK1 has been found to affect cell cycle progression, leading to reduced proliferation, apoptosis, and increased sensitivity to chemo- and radiotherapy [66,67,68].

From a structural perspective, PLK1 contains an N-terminal catalytic kinase domain (KD) and a C-terminal region with two Polo-box domains (PBDI and PBDII), which are involved in the phospho-dependent recognition of substrates and are essential for determining the kinase’s spatiotemporal subcellular localization [69]. In interphase, the KD and PBD establish an inhibitory interaction that suppresses PLK1 catalytic activity. During mitosis, the PBD provides a docking site for proteins with specific phosphorylated motifs [70] and undergoes conformational changes that relieve the inhibition of the KD. This allows the activation loop to be accessed by upstream kinases (such as Aurora-A and its cofactor Bora) for the phosphorylation of the key residue T210, which is necessary for the full functionality of PLK1 [71,72]. In addition to modulating the kinase activity, the phosphorylation status of PLK1 controls its nuclear translocation and its ubiquitin-mediated degradation, which helps to preserve chromosome segregation fidelity and genome integrity [73,74].

**Figure 2:**
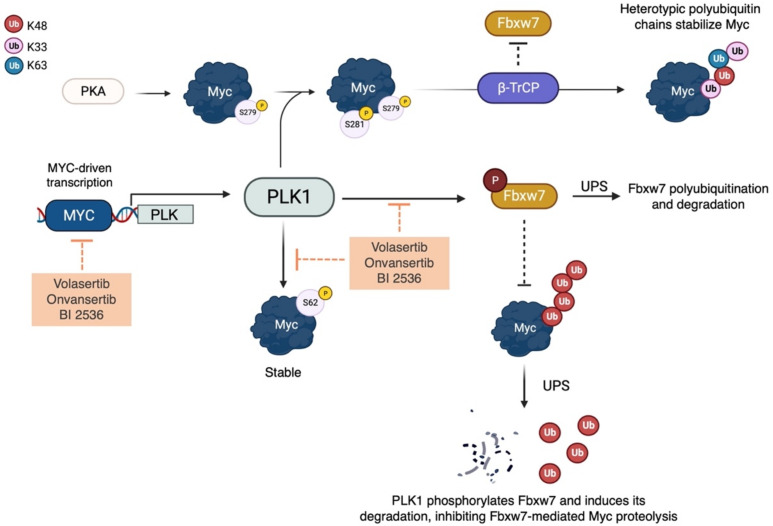
Various mechanisms by which PLK1 stabilizes Myc. PLK1 can directly phosphorylate Myc at S62 or indirectly promote phosphorylation at S281 in a PKA-dependent manner, resulting in the accumulation of a stable, ubiquitinylated form of Myc through β-TrCP binding (right side). Unlike Fbxw7, in fact, β-TrCP assembles K33/K63/K48 heterotypic polyubiquitin chains that do not target Myc for proteasome-mediated degradation. PLK1 also impairs Myc degradation by promoting the proteolysis of Fbxw7, thereby increasing the half-life of Myc. Stabilized Myc, in turn, enhances PLK1 transcription. These mechanisms work together to help maintain high levels of Myc in MYC-amplified tumours, which is associated with poor prognosis. The inhibitors acting on this pathway are shown (see also Section 5.1 for references). Created with BioRender.com (accessed on 20 January 2023).

PLK1 has a complex interactome network involving various pathways beyond mitosis, such as immune response [75], epithelial-to-mesenchymal transition [76,77], and cell death signalling [78,79]. MYC-amplified tumours often have upregulated PLK1, which creates a feed-forward interaction with Myc that maintains high levels of both proteins, predicting poor prognosis [80,81,82]. The main regulatory role of PLK1 is to enhance Myc stability, as seen in the reduced expression of c-Myc upon PLK1 depletion [83]. PLK1 kinase activity is necessary for Myc protein accumulation, through phosphorylation of the key residue S62 [84]. In addition, following the phosphorylation of Myc at S279 by PKA [85], PLK1 can phosphorylate c-Myc at S281, allowing the ubiquitin ligase SCF^β-TrCP^ to bind and ubiquitinylate the oncoprotein. This alternative post-translational event increases c-Myc stability and contributes to Myc-dependent cell transformation [51]. However, the main mechanism of Myc stabilization does not seem to involve the phosphorylation of Myc at S62, but rather PLK1′s ability to affect the activity of Myc-degrading machinery. Evidence from the rescue of N-Myc levels in response to PLK1 pharmacological inhibition by using the proteasome inhibitor MG132 led Xiao and colleagues to propose a possible crosstalk between PLK1 and the E3 ubiquitin ligase Fbxw7; their study showed that wild-type PLK1, but not the kinase mutant K82R, suppresses Fbxw7 activity by promoting its phosphorylation and autocatalytic poly-ubiquitination in MYCN-amplified cellular models [50]. A similar PLK1/Fbxw7/Myc axis was also identified in c-MYC-driven medulloblastoma, with PLK1 antagonizing Fbxw7-mediated c-Myc degradation [86] (Figure 2).

Myc stabilization by PLK1 also plays a prominent role in maintaining the autophagy pathway in tumour cells: knockdown of PLK1 leads to a significant reduction in c-Myc protein levels, impacting on MYC transactivation and impairing Myc-mediated autophagy in osteosarcoma cells [87]. While PLK1 modulates Myc stability, the main contribution of Myc to the oncogenic relationship with PLK1 is through transcriptional regulation. This is supported by the presence of a Myc E-box binding site upstream of the PLK1 transcription start site, and the finding that the suppression of c-Myc leads to a corresponding reduction in both PLK1 mRNA and phospho-T210 protein levels in B lymphoma cells expressing a tetracycline-repressible MYC transgene [50,81]. ChIP experiments performed on neuroblastoma and lymphoma cell lines also demonstrated a significant recruitment of c-Myc to the PLK1 E-box, suggesting that c-Myc directly regulates the PLK1 transcriptional program [50,81]. Based on these findings, targeting PLK1 has the potential to indirectly target Myc-dependent pathways and address the current challenge of developing a therapeutic approach directed against the undruggable Myc proteins.

### 2.2. Aurora-A and Aurora-B Kinases

An intriguing feedback loop with Myc is described for the serine/threonine Aurora kinases. Three members of this family are present in eukaryotes: Aurora-A and Aurora-B, which are involved in the correct execution of mitosis, and Aurora-C, which is mostly implicated in meiosis [88]. Aurora-A and Aurora-B share 71% identity in their kinase domains [89] and have complementary functions in mitotic cells. The Aurora-A protein localizes at centrosomes, where it participates in the maturation and separation processes in the G2 and M phases, facilitating the recruitment of PLK1 onto CEP192 [90]. During mitosis, it interacts with its major activator, TPX2, allowing for the correct formation and orientation of the bipolar spindle [91,92]. On the other hand, Aurora-B interacts with INCENP, constituting the chromosome passenger complex (CPC) with Survivin and Borealin, which ensures chromosome cohesion, the correct attachment of microtubules to kinetochores, and cytokinesis [93,94].

Both proteins are linked to cancer [95,96]. Aurora-A is frequently overexpressed or amplified in a variety of solid and hematologic (blood-related) tumours [89,97], and according to the Cancer Genome Atlas (TCGA), it is overexpressed in almost 88% of the tumours observed [98]. Aurora-A can promote cell transformation when subjected to a suitable cellular background [99]. Additionally, the kinase promotes epithelial-to-mesenchymal transition [100,101], the expression of self-renewal genes in cancer stem cells [102], and cancer cell survival through the regulation of apoptotic modulators (reviewed by [95]). Recently, its oncogenic activities were related to its nuclear localization [54]. On the other hand, the contribution of Aurora-B in cancer development is not well understood, despite the fact that it is upregulated in most aneuploid human tumours [89] and is a poor prognosis factor in hepatocellular carcinoma, non-small cell lung carcinoma, and oral squamous cell carcinoma [103,104,105].

The oncogenic activities of the Aurora kinases have frequently been linked to Myc proteins. In fact, Myc-driven cancers are susceptible to Aurora kinase deprivation or inhibition. One example is c-Myc-driven B-cell lymphoma, in which the overexpression of both Aurora-A and Aurora-B sensitizes cells to pan-Aurora kinase inhibitor treatment [106]. In hepatocellular carcinoma (HCC) cells, Aurora-A interacts with and stabilizes c-Myc, promoting tumour cell survival [107]. In neuroendocrine prostate cancer, Aurora-A and N-Myc interact with each other and drive an oncogenic gene expression program [108]. In neuroblastoma, Aurora-A depletion mimics the effect of N-Myc deprivation in MYCN-amplified (MNA) but not non-MNA cells. This is due to the stabilizing effect of Aurora-A on N-Myc: the kinase binds to the oncoprotein, preventing the Fbxw7 ubiquitin ligase-mediated ubiquitination of Myc [52].

The reason why Aurora kinases’ impairment is an Achilles’ heel of Myc-driven tumours resides both in their importance for cell division, particularly relevant for proliferating cells, and in their ability to regulate Myc transcription and protein stability. Myc-driven expression of Aurora-A and Aurora-B favours mitotic entry and progression, supporting the active proliferation of cancer cells [106,109]. Moreover, the Aurora-A/N-Myc complex, which is observed more frequently in S phase, has been suggested to prevent the formation of replication/transcription conflicts induced by the oncogene’s over-transcription activity [110].

The first physical interaction described between Myc and Aurora family members was the Aurora-A/N-Myc complex in neuroblastoma cells. Depletion of Aurora-A decreases the half-life of N-Myc from 99 min to 55 min in IMR-32 MNA neuroblastoma cells. This Aurora-A function is independent from its kinase activity, since eight different kinase-deficient mutants are able to stabilize the N-Myc oncoprotein [52]. Aurora-A stabilizes N-Myc by protecting it from recognition by the Fbxw7 ubiquitin ligase; moreover, Aurora-A leads to the accumulation of ubiquitinated N-Myc that is non-K48-linked, suggesting that Aurora-A may recruit ubiquitin ligases/deubiquitinases that create a ubiquitinated N-Myc with less degradable ubiquitin chains [52]. Although it is not clear if Aurora-A preferentially interacts with the double-phosphorylated N-Myc (as proposed by [52], whereas [111] did not observe differences between the phosphorylated and unphosphorylated forms for the binding to Aurora-A in vitro), the interplay between Aurora-A and Fbxw7 is well described. In particular, the solution of the crystal structure between the catalytic domain of Aurora-A and the 28–89 N-Myc peptide revealed that Aurora-A binds to the oncoprotein through the MB0 and MBI motifs (residues 61–89) [111]. In vitro pull-down experiments showed that Aurora-A competes with Fbxw7 for the binding of N-Myc within residues 61–89. Nevertheless, the phospho-degron of N-Myc remains able to be recognized by the ubiquitin ligase; thus, a complex involving Aurora-A, N-Myc, and the Fbxw7 is still formed [111].

The region of N-Myc found in the crystal structure is not present in c-Myc [111]. Evidence of Aurora-A/c-Myc binding is controversial, since it was reported that no binding between the two proteins occurs in liver and hepatocellular carcinoma cells [112], while on the other hand, Aurora-A and the double-phosphorylated c-Myc co-immunoprecipitated in TP53-altered (deleted or mutated) HCC cells [107]. However, Aurora-A does play a role in regulating c-Myc expression. It co-immunoprecipitates with the MYCC promoter within the NHE III1 region, which is described to be particularly important for MYCC transcription [112]. Aurora-A has also been shown to act as a co-activator of MYCC transcription in breast cancer [54,113]. Since Aurora-A lacks DNA binding abilities, Zheng and colleagues identified the ribonucleoprotein hnRNP K as the mediator that allows Aurora-A to activate transcription on the MYCC promoter. Depletion of hnRNP K impairs the recruitment of Aurora-A on the MYCC promoter, but not vice versa [54,113]. The transactivation activity of Aurora-A is independent of its kinase activity, as the administration of VX-680 and MLN8237 kinase inhibitors does not affect the expression of the oncogene, in contrast to what can be observed after Aurora-A depletion [113]. hnRNP K also transcriptionally co-activates the p53 protein [114], which negatively impacts the expression of several cell cycle genes, including MYCC [115,116]. Aurora-A may be involved in this process by phosphorylating the ribonucleoprotein at S379, leading to the disruption of its interaction with p53 [115].

On the other hand, c-Myc enhances Aurora-A expression. Mouse fibroblasts with overexpressed c-Myc have a two- to three-fold increase in Aurora-A promoter activity [106]. Using an inducible Myc-ER construct in ChIP experiments, den Hollander and colleagues found that the mouse Aurora-A gene containing two E-boxes is enriched upon induction of c-Myc, suggesting that the oncoprotein directly binds to the Aurora-A promoter, although they were not able to detect the recruitment of c-Myc on either of the Aurora kinase human genes [106]. Lu and colleagues also found a correlation between Aurora-A and c-Myc mRNA in HCC cells, and used ChIP experiments to show that c-Myc binds to the Aurora-A promoter in the highly conserved E-box regions within the CPG islands [112].

In light of their roles in modulating Myc expression and stability, it is interesting to highlight the similarity between the two Aurora kinases. For example, the specificity for the binding of Aurora-A to its activator TPX2 is determined by only one residue, i.e., G198, which is different from that in Aurora-B, N142 [117]. Moreover, the G198N substitution is sufficient to convert Aurora-A into a kinetochore-localized Aurora-B-like kinase [117,118,119,120]. Furthermore, Aurora-A/INCENP binding has also been observed [121]. This evidence suggests a sort of interchangeability between Aurora kinases A and B. However, despite the similarity between Aurora-A/Aurora-B and c-Myc/N-Myc, the interplay with each other is quite different. Aurora-A stabilizes the N-Myc protein [52], and, in turn, high levels of N-Myc directly or indirectly increase Aurora-A mRNA levels (e.g., [122]), establishing a feedback loop that feeds itself. Conversely, Aurora-B does not affect N-Myc protein levels in neuroblastoma [123,124] or retinoblastoma cells [125] upon depletion. However, Aurora-B levels do decrease after N-Myc knockdown [125]. Aurora-B is also transcriptionally activated by direct binding of N-Myc to motifs upstream of the transcription start site [124,125] and by indirect binding of c-Myc in mice, resulting in a 30-fold increase in Aurora-B promoter activity upon Myc-ER activation [106]. It is worth mentioning that Jiang and his colleagues recently identified a novel regulatory mechanism for Aurora B that can stabilize c-Myc through kinase-dependent activity in acute lymphoblastic leukaemia. Experimental results show that Aurora B directly phosphorylates c-Myc at the S67 residue, thereby promoting its stability by counteracting GSK-3-mediated T58 phosphorylation. Notably, sequence alignment did not reveal the S67 phosphorylation site in N-Myc, potentially explaining the non-regulatory role of Aurora B in MYCN-amplified cancers [53].

**Figure 3:**
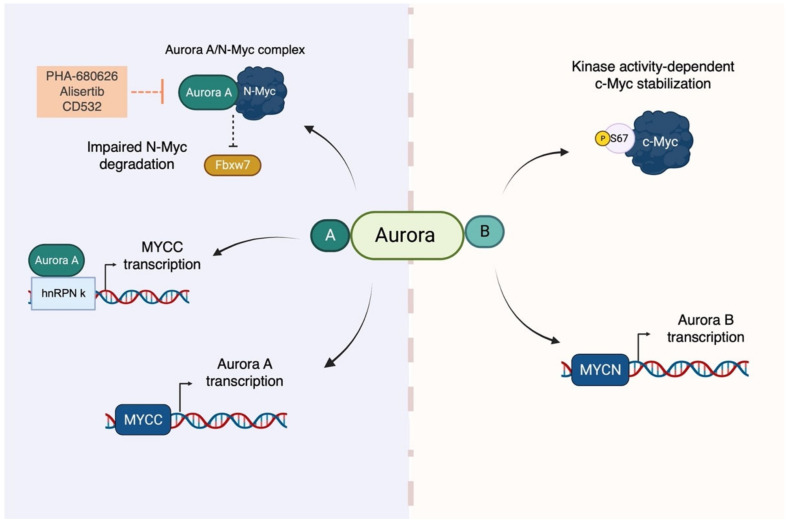
Schematic exemplification of the main functional interplays between Myc and Aurora kinase A (left)/B(right). Aurora-A physically interacts with N-Myc to form a complex that prevents N-Myc from the proteolytic degradation mediated by the ubiquitin ligase Fbxw7. From a transcriptional point of view, Aurora-A contributes to regulating MYCC expression in combination with hnRNP K. Furthermore, Aurora-A is a target gene of MYCC, which enhances Aurora-A transcription. A similar regulatory relationship is described for MYCN and Aurora-B, with MYCN able to bind to motifs upstream of the Aurora B transcription starting site. A kinase-dependent stabilization of Myc involves phosphorylation at serine residue 67, which is a characteristic feature of Aurora kinase B. The inhibitors acting on this pathway are shown (see also Section 5.2 for references). Created with BioRender.com (accessed on 20 January 2023).

In conclusion, the Myc and Aurora family of proteins cross-regulate each other, establishing feedback mechanisms that are particularly relevant for cancer progression (Figure 3). In addition, Aurora-A also impacts the expression and stability of Myc proteins by participating in other processes, such as phosphorylating several members of the PI3K/AKT and Wnt/β-catenin pathways, or proteins involved in Myc stability, such as GSK3β and p53 [101,126,127,128]. It is clear that untangling this intricate network of interactions that affects both the cell cycle and the activity/stability of Myc will need more in-depth investigation.

## 3. Direct and Indirect Myc Regulation by Metabolic Kinases

In order to meet the bioenergetic demand of rapid proliferation, cancer cells must improve their sources of energy. To do this, they put pressure on metabolic pathways that ensure an increase in glucose, angiogenesis, protein synthesis, and DNA synthesis. Several kinases control different key metabolic pathways, such as the Wnt/β-catenin, Notch, MAPK/ERK, and PI3K/AKT pathways, and are considered interesting therapeutic targets. These signalling cascades are also important for the synthesis and stabilization of Myc proteins. Here, two examples of multitasking kinases that regulate Myc proteins are described.

### 3.1. GSK-3

Glycogen synthase kinase-3 (GSK-3) is a ubiquitously expressed and highly evolutionarily conserved serine/threonine kinase that exists in two isoforms encoded by distinct genes, GSK-3α and GSK-3β. GSK-3 paralogs have a bi-lobal architecture, consisting of a large C-terminal globular domain responsible for the kinase activity and a small ATP-binding N-terminal lobe. While GSK-3α and GSK-3β share some common substrates, they are differentially expressed in human tissues and have unique biological roles [129]. Unlike other kinases, GSK-3 is constitutively active under resting conditions and is inhibited by the phosphorylation at key serine residues within the N-terminal domain (S21 for GSK-3α and S9 for GSK-3), which prevents access to the active site by substrates [130]. GSK-3 is involved in several pathways (e.g., PI3K/PTEN/Akt/mTOR, Ras/Raf/MEK/ERK, Wnt/β-catenin, Hedgehog, Notch [131,132]) and plays a decisive role in a wide range of diseases [133,134].

**Figure 4:**
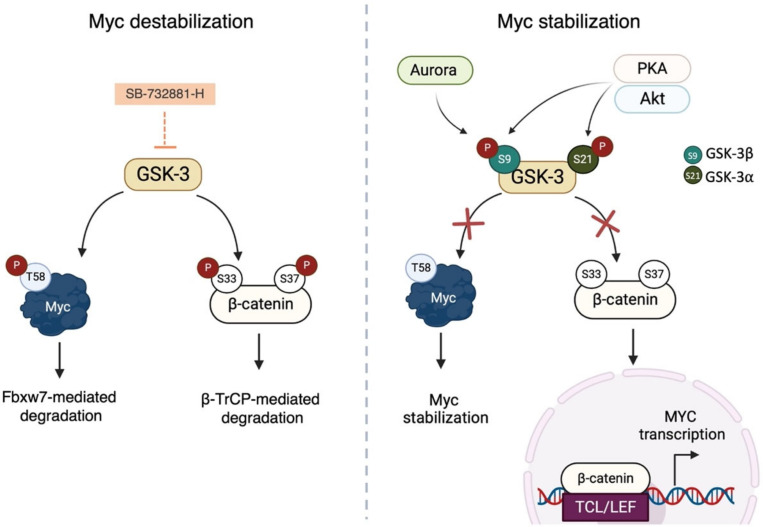
Main Myc regulatory mechanisms by GSK-3. GSK-3 can directly phosphorylate Myc at T58, thus destabilizing the protein and committing it to degradation. In addition, GSK-3 phosphorylates β-catenin and induces its proteolysis, preventing its nuclear translocation. The phosphorylation of GSK-3 at S9/S21 by different kinases prevents both Myc and β-catenin degradation and determines both Myc stabilization, as well as nuclear localization of β-catenin. In the nucleus, β-catenin functions as a coactivator of the TCL/LEF transcription factors and promotes MYC transcription. The main inhibitor acting on this pathway is shown (see also Section 5.3 for references). Created with BioRender.com (accessed on 20 January 2023).

One of the key roles of GSK-3 is in regulating the stability of Myc. The ectopic overexpression of both GSK-3α and GSK-3β was found to increase the phosphorylation of Myc at T58, while the inhibition of the endogenous kinase is associated with a significant reduction in pT58 Myc levels. In addition, there are no significant effects on Myc proteolysis or localization when Myc has a T58A mutation [38,135]. One of the best-characterized tasks of GSK-3 is its role in the destruction of the β-catenin multi-protein complex. β-catenin is a multitasking molecule that is both a core component of the cadherin complex, which coordinates cell–cell adhesion, and a crucial signal transducer in the Wnt pathway. GSK-3, along with Axin and adenomatous polyposis coli (APC), phosphorylates β-catenin at T41, S37, and S33 [136]; phosphorylated S33 and S37 interact with the β-propeller domain of β-TrCP [137], thus priming β-catenin for degradation and ultimately impacting on Myc. Interference with β-catenin proteolysis promotes its translocation into the nuclear compartment [138], where β-catenin acts as a co-activator of the TCF/LEF transcription factors, which control the expression of a wide range of genes involved in oncogenic transformation, including MYC [139] (Figure 4). Indeed, the pharmacological inhibition of GSK-3 has been reported to upregulate both Myc and β-catenin protein levels, thus enhancing Myc-mediated apoptosis in neuroblastoma and KRAS-dependent cellular models [140,141].

GSK-3 exists in different phospho-isoforms and its activity is often negatively modulated by post-translational modifications [142]. Based on this evidence, GSK-3 activation status is key for the regulation of both Myc and β-catenin stability, highlighting the importance of the crosstalk between GSK-3 and other relevant kinases. For example, Aurora kinase A was found to directly bind to and phosphorylate GSK-3β at Ser9 [127], leading to its inactivation. Higher levels of phospho-GSK-3β reduce β-catenin degradation, which accumulates in the nucleus and upregulates its downstream targets, including MYC. Knockdown of Aurora-A has been shown to reduce the amount of nuclear β-catenin in gastric cancer [127], colorectal cancer [143], and glioblastoma cells [102]. In addition to Aurora-A, other relevant Ser/Thr kinases are known to inhibit GSK-3, disrupting substrate-mediated signalling. An analogue modulatory mechanism is reported for PKA, which physically associates with GSK-3 and inhibits both the isoform α and β in a phosphorylation-dependent manner [144]. The cross-regulation between GSK-3 and other kinases also involves the PI3K/Akt signalling pathway and the effector arms of the Ras pathway [145]. When PI3K is activated in response to a wide range of stimuli, it triggers a cascade of second messengers, leading to the activation of Akt kinase, which inhibits both GSK-3α and GSK-3β through reversible phosphorylation at S21 and S9, respectively, derepressing GSK-3 substrates [146]. Blocking PI3K releases GSK from Akt-mediated inhibition, promoting T58 Myc phosphorylation and its subsequent degradation [147,148,149].

The remarkable participation of GSK-3 in a wide range of cellular processes makes this kinase an interesting pharmacological target. However, its multifaceted biological functions do not allow a high degree of inhibition for translation in human diseases to be achieved [133], driving research efforts toward investigating GSK-3–substrate interactions to develop tailored therapies at the level of individual disease.

### 3.2. PKA

The cAMP-dependent protein kinase A (PKA) is considered the prototypical Ser/Thr kinase and regulates many cell signalling events [150,151] such as DNA synthesis, regulation of transcription, and metabolism [152]. It is not surprising that PKA dysregulation is frequently associated with cancer [153], cardiovascular disease [154,155], and neurological disorders [156,157,158]. The kinase is a heterotetramer consisting of two regulatory subunits (R) and two catalytic subunits (C). The cooperative binding of two molecules of cAMP to each R subunit leads to a conformational change that results in the release of the active C subunits [159,160], which can phosphorylate downstream targets. In mammalian cells, two types of R subunits (RI and RII) coexist, with two isoforms of each type (RIα, RIβ, RIIα, and RIIβ). The PKA heterotetramer containing the RI subunit is called PKA type I, while PKA type II contains the RII isoform. The regulatory subunit type confers to the holoenzyme different localization, tissue distribution, and cAMP affinity, ensuring specificity for a large number of substrates, as well as diverse sensitivity to the second messenger cascade. In addition, three isoforms of the C-subunit are described, with the two major isoforms (Cα and Cβ) having multiple splice variants that introduce diversity into the first exon [159].

**Figure 5:**
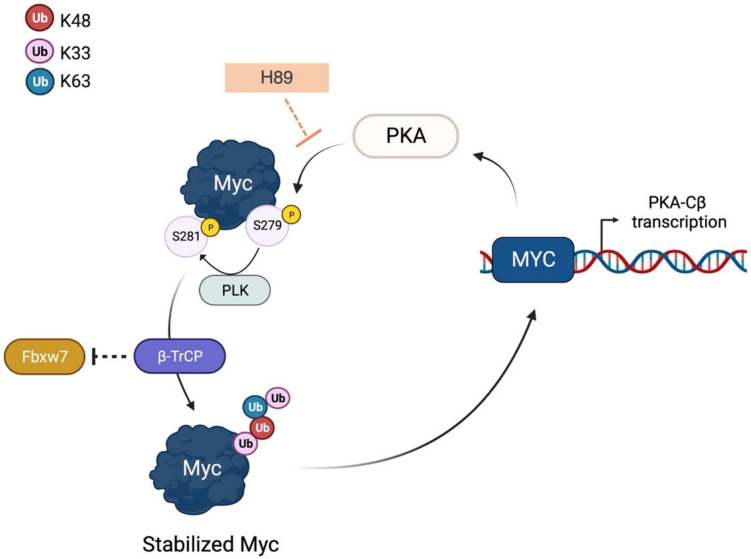
Myc is stabilized via a PKA/PLK1/Myc axis. Myc is stabilized via a PKA/PLK1/Myc axis, which triggers the sequential phosphorylation of the residue S279 by PKA and the residue S281 by PLK1. Stabilized Myc, in turn, enhances the transcription of PKA and, in particular, of the subunit PKA-Cβ, highlighting the relevance of PKA isoform-specific regulatory mechanisms. The main inhibitor acting on this pathway is shown (see also Section 5.4 for references). Created with BioRender.com (accessed on 20 January 2023).

PKA establishes a regulatory loop with Myc, at first stabilizing the oncoprotein, which, in turn, promotes the expression of the kinase. Inhibition of PKA with the specific inhibitor H89 or endogenous pseudosubstrate inhibitor PKI decreases c-Myc protein levels in different cell lines (PC3 and HeLa), an effect that is reversed by the proteasome inhibitor MG132 [85]. c-Myc stabilization is facilitated by PKA-mediated phosphorylation of the oncoprotein at S279, which primes a second phosphorylation mediated by PLK1 at S281 (as described above). PKA is able to phosphorylate ~50% of cellular c-Myc; the non-phosphorylatable S279A mutant is not destabilized by H89 treatment and impairs PLK1-mediated phosphorylation, suggesting a prominent role for this phosphorylation site in c-Myc protein stability [85]. c-Myc protein stability is also governed by a fine balance between ubiquitination events. The βTrcp ubiquitin ligase counteracts Fbxw7-mediated degradation of c-Myc by linking non-degradable K-Ub [51]. c-Myc recognition by βTrcp is ensured by PLK1 phosphorylation in the N-terminal of the oncoprotein sequence [51], highlighting the key influence of the PKA-PLK1-βTrcp axis on c-Myc stability (Figure 5).

Despite the clear role of PKA in aiding Myc protein stability, knockdown of PKA demonstrates isoform-specific regulation: siRNA against PKACα induces an increase in c-Myc mRNA and protein, whereas knockdown of PKACβ leads to a slight decrease in c-Myc protein and no mRNA enrichment [85]. PKACβ is overexpressed in rapidly proliferating prostate cancer cells, while PKACα overexpression was reported to induce trans-differentiation of LNCaP cells into neuroendocrine-like cells [85]. The feedback between PKACβ and Myc is well described. c-Myc transcriptionally activates PKACβ in pancreatic cancer. Wu and colleagues identified two non-canonical E-boxes within intron 1 and in the 5′ region flanking intron 1. Luciferase experiments demonstrated that c-Myc can bind the 5′/intron 1 region and activate luciferase expression four- to six-fold, comparable to other classical Myc targets [161]. PKACβ expression is abrogated upon c-Myc mutations in the TAD domain or defects in the interaction with MAX. Conversely, the role of PKACα in c-Myc stability is controversial: it is also a key regulator of the CREB transcription factor, which, together with p300, inhibits Myc expression [162,163]. Additionally, among PKACα (but not PKACβ) substrates, ENO1 and MBP1, which are splice variants described as Myc transcriptional repressors, have gained attention [85]. Some studies have observed increased levels of c- and N-Myc in cAMP stimulation conditions, while PKACα siRNA attenuates phosphorylation of its substrates and progressively decreases c- and N-Myc protein levels [55].

The overall data suggest that PKA isoforms, due to their differential substrate selectivity, have opposite effects: the Cα subunit indirectly reduces the expression of the oncogene through the activation of Myc transcriptional repressors [85,164]. At the same time, inhibition with pan-PKA inhibitors stabilizes the Myc protein, which, in turn, promotes the expression of the Cβ subunit, protecting Myc from proteasome-mediated degradation [85]. The differential cell responses to the diverse activities of PKA isoforms highlight the complexity of the cross-regulation between PKA and Myc. Indeed, Myc regulation is influenced by the cellular availability of interacting kinases, which is often dependent on tissue-specific expression, environmental conditions, or protein localization. For example, PKAI is mostly cytoplasmic and its activity is associated with cell growth and proliferation, while PKAII is anchored to subcellular structures and compartments and is mostly associated with differentiation [159,165]. This scenario emphasizes once again the concept that the activity of Myc in cells results from a very delicate balance of time- and space-regulated phosphorylation cascades.

## 4. Direct and Indirect Myc Regulation by Histone-Associated Kinases

Histone-associated kinases are able to phosphorylate and modify the histone proteins packaging DNA within the nucleus and can regulate various processes in the cell, including gene expression, DNA replication, and DNA repair, by modifying the structure and accessibility of the underlying DNA. PIM and BRD4 are examples of histone-associated kinases, for which we will describe the cross-regulation with Myc.

### 4.1. PIM

The PIM (proviral integration site for Moloney murine leukaemia virus) family of serine/threonine kinases consists of three highly similar isoforms designated as PIM-1, PIM-2, and PIM-3, which share functional redundancy and overlapping specificity for a wide range of substrates involved in cell proliferation and survival [166,167]. PIMs are overexpressed in several human haematological malignancies as well as solid tumours, frequently correlating with advanced clinical stages and poor prognosis [168,169,170,171]. In contrast to other protein kinases, PIMs lack a regulatory domain and adopt a constitutively active conformation that is maintained via the establishment of a salt bridge between acidic residues in the A-loop and a conserved arginine residue in the C-loop, keeping the ATP binding pocket open and ready for catalysis [172,173].

Although the three members of the PIM kinase family have been classified as weak oncogenes and are not sufficient alone to promote the tumour transition toward a more aggressive form, their potential is exploited through the synergistic interplay with other proteins. The cooperative partnership between PIMs and Myc is well-characterized in different types of cancers, with PIMs widely reported to play a key role in regulating Myc stability and transcriptional program [174,175,176,177]. PIM overexpression is linked to c-Myc stabilization in vivo through a phosphorylation-dependent mechanism that occurs with an isoform-specific efficiency. As described by Zhang and colleagues, PIM-1 and PIM-2 stabilize c-Myc almost to the same extent and are directed against the same phosphorylation sites, but PIM-1 preferentially phosphorylates c-Myc at S62, whereas PIM-2 is more efficient in the direct phosphorylation at S329 residue, contributing to increasing c-Myc half-life. Remarkably, phospho-S329 also plays a prominent role in enhancing c-MYC transcriptional activity [56]. Knockdown of PIMs results in a dramatic reduction in endogenous c-Myc protein levels, with a major downregulation achieved by simultaneously silencing PIM-1, -2, and -3 [178]. Interestingly, the expression of PIM-3 alone was shown to overcome some defects arising from the depletion of PIM-1 and PIM-2: PIM-3, in fact, enhances cap-dependent translation in triple-knockout mouse embryonic fibroblasts and upregulates c-Myc levels without affecting protein stability. This evidence suggests that PIM isoforms may mutually regulate each other either directly or through substrate competition [179]. Additionally, PIMs critically supervise the balance between anti- and pro-apoptotic factors in Myc-driven tumorigenesis. MYC-amplified cells, in fact, exhibit a high proliferation rate and are prone to apoptosis; notably, PIM kinases, especially PIM-2, can phosphorylate the pro-apoptotic proteins BAD and Bcl-2, thereby counteracting their function and sustaining cell proliferation [180,181].

**Figure 6:**
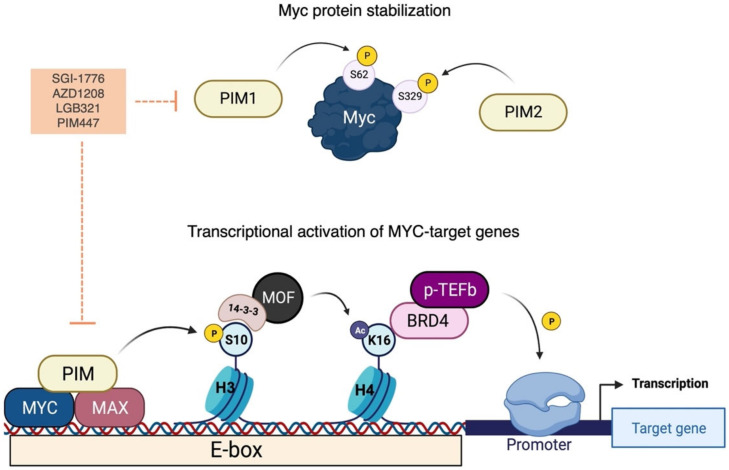
Regulation of Myc by PIM kinases. PIMs phosphorylate Myc at different residues with an isoform specificity and contribute to enhancing protein stability. In addition, PIMs contribute to epigenetic modulation by forming a ternary complex with MYC-MAX, thus regulating the expression of genes under MYC transcriptional control. PIM, in fact, phosphorylates the histone H3 within the E-box element of MYC-dependent genes at the serine residue 10. Phosphorylated H3 recruits the phosphoserine-binding protein 14-3-3, which interacts with the histone acetyltransferase MOF. MOF, in turn, catalyses the acetylation of the histone H4 at K16. Acetylated histone H4 provides a binding platform for BRD4 and p-TEFb, facilitating transcriptional elongation. The inhibitors acting on this pathway are shown (see also Section 5.5 for references). To simplify, dual inhibitors are not reported in the figure. Created with BioRender.com (accessed on 20 January 2023).

However, one of the most important mechanisms that contribute to the oncogenic cooperation between Myc and PIM-1 is the dynamic modulation of gene transcription through epigenetic mechanisms. PIM-1 can form a ternary complex with the MYC-MAX heterodimer, which allows PIM-1 to phosphorylate S10 of histone H3 within the E-box element of Myc target genes. This contributes to the activation of a subset of genes that are under the transcriptional control of Myc [57]. The phosphorylation of H3 by PIM-1 recruits the phosphoserine-binding protein 14-3-3 to the enhancer; the interaction between 14-3-3 and the histone acetyltransferase MOF leads to the acetylation of histone H4 at residue K16 [182]. The interaction between H3S10ph and H4K16ac creates a nucleosome platform for the binding of the bromodomain protein BRD4 (see below); BRD4 stimulates the kinase activity of the positive elongation factor b (p-TEFb), which phosphorylates the C-terminal domain of RNA polymerase II promoting transcriptional elongation [182,183]. In triple-negative breast cancer models, PIM-1 knockdown results in reduced phosphorylation of c-Myc at S62 and histone H3 at S10, as well as a decrease in the total amount of Myc protein. The loss of PIM-1 mRNA after silencing is also reflected in the MYC gene signature, with 11 out of 24 MYC-target genes downregulated in cells silenced for PIM1. A large-scale gene expression profiling in HEK-293 cells that were silenced for either MYC or PIM-1 revealed that PIM-1 contributes to the regulation of 207 of the 1026 MYC-target genes, demonstrating its significant impact as a Myc-dependent chromatin modifier [184,185] (Figure 6).

Interestingly, and as previously discussed for other kinases involved in Myc regulation, the synergy between Myc and PIM kinases is reciprocal and relies on the creation of a feed-forward loop. For instance, Myc-driven lymphoma cells from transgenic mice and human patients exhibit elevated mRNA levels of the isoform PIM-3. The analysis of the nucleotide sequence of the PIM-3 locus revealed a conserved E-box that can be bound by Myc [186]. In addition, PIM-3 enhances 5′-cap-dependent translation, increasing c-Myc levels without affecting protein stability [179]. As further support for the interplay between Myc and PIM, a recent study examined their involvement in ribosomal stress, proposing a novel role for PIM-1 in prostate cancer progression. Among its diverse biological functions, PIM-1 participates in the assembly of the ribosomal subunit 40S by regulating the expression of ribosomal small subunit proteins (RPSs), especially RPS7. Luciferase-based assays indicate that the promoter of RPS7 can be directly bound by c-Myc, confirming the existence of a PIM1-c-Myc-RPS7 axis responsible for abnormal ribosomal biosynthesis and subsequent ribosomal stress [187]. Considering the pivotal role of PIM kinases in Myc regulation, their value as potential targets for the development of new drug candidates deserves further investigation.

### 4.2. BRD4

BRD4 is a bromodomain and extra terminal (BET) protein, showing both acetyl-transferase activity (HAT) and kinase activity. BRD4 dysfunctions are associated with multiple diseases such as cancers, neuro-degenerative disorders, autoimmune diseases, and heart, kidney, lung, and inflammatory diseases [188,189]. BRD4 has been described to be a transcription coactivator and recruiter of chromatin remodelling factors, and it participates in mitosis, pause release of PolII, RNA splicing, and DNA damage and stress responses, most of which are functions in common with Myc [190].

BRD4 is composed of two bromodomains (BD1 and BD2) organised in tandem and an extra terminal domain (i.e., ET, important for its interaction with a wide range of proteins) at the N-terminus, as well as Motif A, Motif B, basic residue interaction domain (BID), and the Ser/Glu/Asp-rich region (SEED) [190]. In contrast, the C-terminus, which hosts the HAT catalytic domain, is an intrinsically disordered region (IDR) [190,191] and is present only in the longer of the two cellular BRD4 isoforms [192]; the disordered structure of this region ensures the flexibility required for the binding of different proteins, i.e., c-Myc [58].

BRD4 and c-Myc co-immunoprecipitate from HeLa nuclear extracts and show a strong nuclear interaction signal when examined using *is*PLAs [58]. To identify the region of interaction between the two proteins, Devaiah and colleagues performed pull-down experiments using recombinant BRD4 and c-Myc truncated proteins [58]. The TAD domain of c-Myc (residues 144-163) interacts with the C-terminus of BRD4 (aa 823-1044), but this interaction is independent of the HAT domain. The BRD4/c-Myc interaction is essential for c-Myc protein stability. In fact, the bromodomain protein phosphorylates c-Myc at T58, leading to its ubiquitination and degradation [58]. MEF cells derived from mice homozygous for floxed BRD4 alleles show decreased levels of pT58 and increased levels of c-Myc protein, whereas overexpression of BRD4 increases phosphorylation at T58 and decreases total c-Myc. Notably, in contrast to GSK-3β, BRD4 is able to phosphorylate T58 regardless of previous phosphorylation at S62, and the bromodomain protein immunoprecipitates with both T58A and S62A c-Myc constructs transfected into HeLa cells [58].

**Figure 7:**
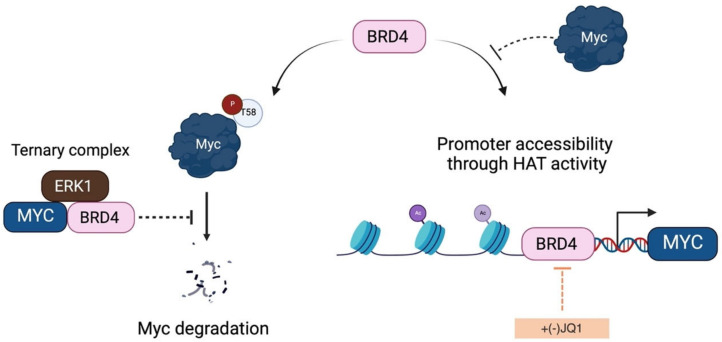
BRD4-dependent regulation of Myc. BRD4 directly phosphorylates Myc at T58 through its kinase activity, thus inducing its degradation and reducing its inhibitory effect on BRD4 histone acetyltransferase activity. However, the physical interaction of Myc and BRD4 with ERK1 to form a ternary complex prevents Myc from BRD4-mediated destabilization. Furthermore, BRD4 promotes MYC transcriptional activation by decondensing the chromatin around the MYC gene by means of its histone acetyltransferase activity. The main inhibitors acting on this pathway are shown (see also Section 5.6 for references). To simplify, dual inhibitors are not reported in the figure. Created with BioRender.com (accessed on 20 January 2023).

The complexity of the interaction between BRD4 and Myc is not limited to the binding of Myc to BRD4′s C-terminus. BRD4 also binds the N-terminal region of the ERK1 kinase, which is responsible for stabilizing Myc through phosphorylation at S62, forming an ERK1–BRD4–Myc complex. Each of these proteins interacts with the others. Notably, ERK1 binds the region containing the kinase domain of BRD4, inhibiting its kinase activity and, therefore, its autophosphorylation and its ability to phosphorylate c-Myc [58]. This helps stabilize c-Myc and prevent its degradation, mediated by BRD4 (Figure 7). Another condition in which BRD4 is unable to phosphorylate Myc is when the bromodomain protein is bound to chromatin, due to overlap of the BD2 domain with the kinase domain [191]. However, the trimeric complex is present in both chromatin-bound and unbound fractions of the nucleus, indicating regulation at various levels. BRD4 is a Myc transcriptional activator and contributes to the expression of Myc at the end of mitosis [193,194,195]. Through its HAT activity, BRD4 promotes the promoter accessibility [196]. While c-Myc has no effect on BRD4 kinase activity, it does influence BRD4′s ability to remodel chromatin by inhibiting its HAT activity [58,190]. Transiently transfected HeLa and U2OS cells with a c-Myc vector show reduced levels of H3K122ac by more than 40% [58]. Notably, when c-Myc is phosphorylated by both BRD4 and ERK1, it fails to inhibit BRD4 HAT activity. Indeed, this activity is dependent on phosphorylation on T58, since the T58A non-phosphorylatable mutant markedly inhibits H3K122ac [58].

From what has been discussed so far, it appears that the regulation of the interaction between BRD4 and Myc is a complex process. BRD4 activates the transcription of Myc by remodelling the chromatin around the Myc gene locus, through its histone acetyltransferase (HAT) activity. At the same time, BRD4 phosphorylates c-Myc on T58, causing its degradation, which reduces the inhibitory effect of c-Myc on BRD4′s HAT activity. In this context, ERK1, which interacts with the BRD4 kinase domain, counteracts the destabilizing effect of BRD4 on c-Myc. This intricate cross-regulation is important for the transcriptional activity of Myc. The degradation of c-Myc interferes with the acetylation of histones at Myc target genes, indicating the need to turn over c-Myc in order to ensure their transcription [196]. It has also been shown that the degradation of Myc is necessary for the pause release of RNA polymerase II [197]. Devaiah and colleagues suggest that the degradation of c-Myc induced by BRD4 influences the pause release of RNA polymerase II, and that the reduction in c-Myc protein levels is balanced by the activation of c-Myc transcription by BRD4 [58].

## 5. Kinase Inhibitors and Myc Proteins

Myc is deregulated and overexpressed or hyperactivated in the vast majority of human cancers. Unfortunately, because of its intrinsically disordered structure, Myc lacks a targetable binding pocket and is, thus, considered undruggable. Thus, the inhibition of Myc oncogenic functions may rely on protein–protein interaction (PPI) inhibitors; however, Myc takes on a secondary and tertiary structure only when in complex with one of its partners, making the identification of PPI inhibitors challenging [198]. Several efforts have been made in order to design disruptors of the Myc/MAX interaction, which is necessary for the Myc oncogenic activities. These PPI inhibitors are small molecules (e.g., IIA6B17 and IIA4B20 [199]; 10074-G5 and 10058-F4 [200]; Mycro1 and Mycro2 [201] and their derivatives) and cell-penetrating peptide inhibitors (Omomyc [202,203]; IDP-410 [204], even though the complete impairment of the binding of Myc on DNA is challenging, and they are found to non-specifically target other transcription factors (such as Jun). Nevertheless, an optimized Omomyc is undergoing phase I/II clinical trial (Omo-103, NCT04808362). However, the Myc pleiotropic activities might be better targeted acting at different levels.

Therefore, another potentially valid approach against MYC-driven cancers is to target the kinases that support the Myc oncogenic functions. Kinase inhibitors are widely used in cancer therapy. It is estimated that one quarter of pharmacological efforts are focused on kinase inhibitor development [205]. All the kinases described above are found to be associated with cancer, and the most promising drug candidates with reported effects on Myc are reported in Table 1.

### 5.1. PLK1 Inhibitors

As previously mentioned, PLK1 constitutes an appealing therapeutic target for overcoming the challenging development of pharmaceutical-grade inhibitors of the Myc protein. PLK1 inhibitors can be grouped into two classes: ATP competitors, which target the binding site of the kinase domain [229], and non-ATP competitors, which target the PBD domain [230]. Although ATP competitors have low selectivity, which can increase the risk of toxic side effects, they generally exhibit good drug-like properties. Four major PLK1 inhibitors belonging to this class have reached clinical trials: BI2536, volasertib (BI6727), onvansertib (NMS-1286937), and GSK461364.

BI2536 is a dihydropteridinone derivative that can inhibit PLK1 at nanomolar concentrations in preclinical experiments with an acceptable safety profile. However, BI2536 had no significant effects on patients with relapsed/refractory solid tumours enrolled in phase II studies [231,232], leading to the discontinuation of its development in favour of volasertib. Volasertib was developed by modifying the structure of BI2536, and has improved physicochemical and pharmacokinetic properties, including a higher distribution volume and prolonged half-life. In addition, volasertib potently inhibits PLK1 (estimated IC_50_ of 0.87 nM in cell-free in vitro assay) without affecting related kinases at concentrations up to 10 µM [233]. Volasertib underwent phase I/II clinical trials for the treatment of solid tumours and haematological malignancies; although the monotherapy seemed promising, it only showed modest antitumour effects [234], and a more complete response was achieved when volasertib was tested in combination with other therapeutic agents [235]. In MNA tumours, the inhibition of PLK1 by volasertib is correlated with a decrease in Myc protein levels. Volasertib reduces c-Myc phosphorylation at S62 and is linked to impaired cell viability as well as PARP cleavage in aggressive T-lymphoma and B-lymphoma cells [81,83]. The effects on MYC transcriptional levels may be due to the mild effect of volasertib on the BET protein BRD4 [236]. This evidence is consistent with the finding that PLK1 inhibition by volasertib leads to the stabilization of the ubiquitin ligase Fbxw7 and the degradation of its downstream target N-Myc in MYCN-overexpressing neuroblastoma cells. In the same cellular model, the combination of volasertib with the Bcl12 inhibitor ABT199 selectively induced apoptosis, suggesting that the Volasertib/ABT199 synergism may be a potential therapeutic strategy [50]. In the same study, similar effects on Myc protein levels were observed with the thiophene-amide inhibitor GSK461364. However, the impact of GSK461364 on Myc is not yet well understood, as phenotypic effects from PLK1 inhibition, which are not dependent on MYCN amplification status, have been reported in neuroblastoma cell lines [237].

The pyrazoloquinazoline derivative onvansertib is an oral and highly selective third-generation ATP-competitive PLK1 inhibitor with an IC50 of 36 nM [238] currently under phase I clinical study for solid tumours [239]. onvansertib was found to suppress tumour growth in both in vivo and in vitro models. In MYC-driven medulloblastoma, onvansertib caused a significant downregulation of MYC-target genes and a substantial decrease in Myc protein abundance. Additionally, PLK1 depletion in response to onvansertib stabilizes the ubiquitin ligase Fbxw7, resulting in a decrease in Myc protein in xenograft models [207]. These data are consistent with the PLK1/Fbxw7/MYC signalling axis described by Xiao and colleagues, and highlight the potential of PLK1 inhibitors as a therapeutic option for the treatment of MYC-overexpressing cancers.

### 5.2. Aurora-A Inhibitors

Aurora-A is the best-described kinase for targeting Myc. Aurora-A and Aurora-B kinase inhibitors are being evaluated in clinical trials for solid and hematopoietic tumours [240]. In addition, Aurora-A has been described as a critical target for MNA neuroblastoma cells. The discovery of Aurora-A’s stabilizing effects on N-Myc through the physical interaction of the two proteins [52] has led to intense research aimed at destabilizing this interface. It is noteworthy that this interaction does not depend on Aurora-A kinase activity; therefore, classical ATP-competitive kinase inhibitors are not useful for disrupting the kinase/N-Myc complex. A few Aurora-A orthosteric inhibitors, however, have been shown to also act as amphosteric regulators, stabilizing Aurora-A in a conformation that is incompatible with N-Myc binding; for this reason, these compounds are hailed as conformation-disrupting (CD) inhibitors. The two best-characterized CD inhibitors are Alisertib (MLN8237) [123,241] and CD532 [208]. Recently, our group identified PHA-680626 as a novel potential CD inhibitor [210]. These compounds induce strong cell death in MNA compared to non-MNA neuroblastoma cells due to the decrease in N-Myc protein levels caused by the impairment of the Aurora-A/N-Myc complex formation.

The conformation of Aurora-A bound to CD inhibitors, which allows the disruption of the oncogenic complex, is characterized by the opening of the N- and C-lobes of the protein [242] and the flipping of the activation loop (residues 274–293) into a closed conformation. The conserved DFG motif (residues 274–276) is crucial for Aurora-A activity, and can assume two main conformations: the active DFG-In and the inactive DFG-Out. It was originally thought that the DFG-In state was strictly associated with the open conformation, but it has since been shown that CD532, a known Aurora-A CD inhibitor, stabilizes the protein in a closed conformation while maintaining the DFG-In state [208]. In fact, recent work has questioned the CD ability of a panel of kinase inhibitors and, using Förster resonance energy transfer (FRET) methodology, has revised the classification of known CD inhibitors [243].

Recently, the protein targeting chimeras (PROTAC) methodology has been explored for Aurora-A, with promising results [244,245,246]. Tang and colleagues developed a PROTAC from a ribociclib (CDK4/6 inhibitor) scaffold, which is able to target the Aurora-A/N-Myc complex [246]. It is worth noting that the idea of cellular pools of Aurora-A that interact in a spatially and temporally regulated manner with several partners [54] is gaining attention. In line with this idea, centrosome-localized Aurora-A is not sensitive to an MLN8237-based PROTAC (PROTAC-D)-mediated degradation [245]; moreover, deprivation of the kinase following PROTAC JB170 administration results in the accumulation of cells in the S phase, while kinase inhibition leads to G2/M enrichment in leukaemia and neuroblastoma cell lines [244], suggesting a need for further investigation into Aurora-A’s non-mitotic functions.

### 5.3. GSK3 Inhibition

Over the last decades, GSK-3 inhibitors have been extensively investigated as potential pharmacological tools against neurodegenerative diseases and psychiatric disorders, but the well-established involvement of GSK-3 in oncogenic pathways suggests its therapeutic relevance for the development of novel anticancer therapies. Even though some compounds have reached clinical trials, most of them did not satisfy the endpoints and were discontinued due to safety issues. Currently, the most promising GSK-3 inhibitor is the compound 9-ING-41 (elraglusib), which is under clinical evaluation for the treatment of several cancers in combination with other chemotherapeutic agents (NCT03678883, NCT05239182).

One of the main concerns about the clinical application of drug candidates directed against GSK-3 still remains the controversial effects of GSK-3 inhibition, which leads to the stabilization and oncogenic activation of β-catenin. Surprisingly, the stabilization of both β-catenin and c-Myc observed in response to the dual GSK-3α/3β inhibitor SB-732881-H correlates with a paradoxical increase in c-Myc-mediated apoptosis in preclinical models of KRas-dependent human cancers, presenting a new perspective on the therapeutic implications of GSK-3 inhibitors [141]. However, a high degree of inhibition of GSK-3α/3β is mostly associated with unacceptable toxicity due to the low selectivity of available compounds. For this reason, Wagner and colleagues rationally design paralog-selective inhibitors of GSK-3 able to discriminate between the two existing isoforms. Notably, the inhibition of GSK-3α kinase function did not stabilize β-catenin in acute myeloid leukaemia cells, providing a feasible strategy to develop paralog-selective GSK-3α inhibitors enforceable to cancer therapy [247].

### 5.4. PKA Inhibitors

Due to its involvement in a wide range of signalling pathways, PKA dysregulation is often associated with cancer [153]. As described before, the regulative subunits of PKA are also differentially expressed in cancer, with the RIα upregulated in a series of neoplasms, whereas the RIIβ inhibits tumour growth. Another level of complexity is introduced by the AKAP, which controls the localisation of PKA near its substrates [165]. Thus, the inhibition of PKA represents a great challenge. PKA inhibitors can be divided into three main categories: ATP antagonists, cAMP antagonists, and the protein kinase inhibitor peptide (PKI).

ATP antagonists that inhibit PKA, such as H89 and KT5720, present one key issue: their IC50 depends on ATP concentration, which can vary greatly inside the cell. In addition, both H89 and KT 5720 have been shown to inhibit other kinases at similar or even lower concentrations to those used to inhibit PKA [248]. H89 has been shown to enhance the cytotoxic effects of the anticancer agent tetrandrine in several cancer cell lines. Cells with higher levels of c-Myc were more sensitive to this treatment combination, and cells resistant to treatment could be sensitized by c-Myc overexpression. These findings were confirmed in a MDA-MB-231 breast cancer murine xenograft tumour model, in which combined therapy showed a significant reduction in tumour weight compared to single treatments. These data suggest that this treatment combination could be effective for tumours expressing high levels of Myc [249]. In addition, H89-mediated inhibition of PKA has been shown to decrease the level of Myc protein in several prostate cancer cell lines due to the impairment of PKA-mediated stabilization of Myc [85].

PKI is the endogenous pseudosubstrate inhibitor of PKA. Its amino acid sequence mimics the inhibitory subunit of the kinase, impeding the binding of cAMP to the PKA catalytic subunit [250]. There are three isoforms of PKI (α, β, γ), which differ in expression pattern and Ki. PKI is more specific for PKA than ATP-competitive inhibitors, but as a peptide, it is less stable than small-molecule inhibitors and cannot pass the cell membrane. Synthetic peptide analogues have been developed, among which is PKI-(6-22)-amide, which has, however, been shown to reduce the toxicity of taxol and taxane in prostate cancer cells [251,252]. In addition, overexpression of PKI has been demonstrated to cause a shift in downstream regulation, increasing ERK phosphorylation following cAMP signalling, and the gene for PKIα has been found to be overexpressed in several cancer types. In DU145 prostate cancer cells, PKIα depletion with shRNA resulted in reduced migration and reduced tumour volume in murine xenografts [253]. PKI has been shown to decrease the stability of the Myc protein in prostate cancer, as observed for H89 [85].

Since several cAMP targets are present in the cell, cAMP analogues such as Rp-cAMP interfere with pathways that only partially overlap with those of other PKA inhibitors. As far as we know, no studies on the correlation between Rp-cAMP and Myc levels have been reported yet, but Rp-cAMP-induced PKA inhibition has been shown to enhance the stimulating effects of prostaglandin E2 on glioblastoma cell lines [254]. Considering the high degree of cAMP and PKA regulation and the ramification of their downstream effects, it should not be surprising that the exact effects of PKA inhibition have yet to be understood.

### 5.5. PIM Kinases

PIM inhibitors have shown in vitro activity through both Myc-dependent and Myc-independent pathways against numerous tumour cell lines ranging from haematological tumours to breast and prostate cancer [31,177,178,211,214,215], but their results in clinical studies are often disappointing.

SGI-1776 is an ATP-competitive imidazo [1,2-b]pyridazine small molecule highly selective for all three PIM isoforms, with inhibitory concentration values in the nanomolar range and without any relevant effects on cell cycle kinases. At cellular levels, SGI-1776 exhibited promising biological activity, and the SGI-1776-mediated inhibition of PIMs resulted in the reduction in phospho-S62 Myc and total Myc protein abundance in chronic lymphocytic leukaemia cells [212]. Consistent with these findings, SGI-1776 decreased phospho-S62 Myc and impaired the MYC-driven transcriptional program in mantle cell lymphoma models too [213]. Although this compound also shows evident effects in models of breast cancer and diffuse large B-cell lymphoma cell lines (DLBCL), it showed not insignificant cardiotoxic effects in a phase I study (NCT01239108), which was then terminated.

In the landscape of PIM inhibitors, an interesting candidate is NVP-LGB321. LGB321 is a potent ATP-competitive inhibitor able to bind to and prevent the activation of all the three PIM isoforms. Preclinical data demonstrated the capability of LGB321 to reduce phospho-S62 c-Myc levels, whereas no substantial effects were observed on MYC mRNA, suggesting that PIM regulates Myc via post-transcriptional mechanisms [211]. LGH447 (PIM447) is a pan-PIM inhibitor derived from the tool compound LGB231, and although it gave promising results against multiple myeloma in clinical studies, current data suggest that the true potential of this inhibitor relies upon its ability to enhance the efficacy of other anticancer drugs. Paìno and colleagues, in fact, highlighted the synergistic anticancer effects of PIM447 on a panel of different myeloma cell lines and observed a marked decrease in the phosphorylation of c-Myc at S62 and a downregulation of MYC mRNA levels [216]. Another compound, AZD1208, is an orally available and potent drug able to inhibit all three isoforms. It induced cell cycle arrest and cell death in acute myeloid lymphoma cell lines, as well as being active against prostate cancer and acute myeloid leukaemia (AML) xenograft mice models, but lacked clinical efficacy in phase I studies. Even if AZD1208 performed poorly in clinical trials, it was able to enhance the sensitivity to radiation of Myc-CaP cells and of Myc-CaP tumours in nude mice. The effectiveness of this therapy combination might lie in the fact that Pim1 is a pro-survival kinase that is upregulated in response to stressors such as radiation; PIM inhibition might then be crucial to block survival of irradiated cells. At the same time, AZD1208 downregulates p53, which is instead upregulated upon radiation.

Recently, SEL24/MEN1703 has been identified as a first-in-class dual PIM/FLT3 inhibitor and is currently under clinical evaluation for the treatment of acute myeloid leukaemia. SEL24/MEN1703 demonstrated on-target activity as a pan-PIM inhibitor, inhibiting the cell proliferation of various diffuse large B-cell lymphoma (DLBCL) cell lines and DLBCL tumour growth in murine xenograft models. Although the biological effects of SEL24/MEN1703 are not entirely dependent on its activity on Myc, there are at least three major mechanisms attributed to Myc inhibition: PLK1 inhibition, CD20 expression, and CD47 suppression. As previously discussed, PLK1 is a crucial player in Myc stabilization and regulation, and, therefore, the decrease in PLK1 following SEL24/MEN1703 administration may be crucial in thoroughly suppressing Myc-related pathways. CD20 expression was speculated to enhance the activity of anti-CD20 antibodies, and is regulated by the PIM-Myc axis; MYC, in fact, represses the gene encoding CD20. Based on these considerations, the combination of SEL24/MEN1703 and rituximab (an anti-CD20 antibody, which is the standard of care for this type of tumour) resulted in higher complement-dependent toxicity in DLBCL cells compared to rituximab alone. Indeed, increased CD20 surface expression occurred upon Myc downregulation, as well as enhanced transcription of CD20 that was not observable in cells modified to express the T58A Myc mutant. In addition to the increased expression of CD20, the action of rituximab was also enhanced by the Myc-dependent depletion of CD47, a phagocytosis inhibitory signal. These Myc-dependent effects were also shown to be true for other PIM inhibitors such as SGI-1776 and PIM447 [178,216].

In conclusion, the extensive overlapping of PIM kinase phosphorylation patterns with the targets of other kinases has, so far, made it challenging to develop effective treatments through PIM inhibition alone; combined therapies might be the turning point for the application of PIM inhibitors.

### 5.6. BRD4 Inhibitors

BRD4 inhibitors are also widely used to treat a variety of cancer-related and non-related disorders [188,255]. Bromodomain inhibitors (BETis) are small molecules designed to mimic acetyl-lysine, which is recognized by BRD4 through its BD domains, thereby preventing interactions between BRD4 and chromatin [256]. Given the strong correlation and cross-regulation between BRD4 and Myc, BETis are effective against Myc-driven cancers; indeed, Myc is also used as a readout to predict tumour responsiveness to BETis [257].

It is worth noting that cancer cells can acquire resistance to BETis. Cancer cells compensate for the loss of bromodomain transcriptional activity by upregulating BRD4 downstream pathways, such as Wnt/β-catenin, Hedgehog, and RAS-RAF-MAPK (reviewed by [188]), which restore Myc expression. This highlights the need for other types of interventions or combinatorial drug administration.

To improve the efficacy of BETis, they have been tested in combination with inhibitors of PI3K/AKT [258] and MAPK/ERK1-2 (MEKi) [259] signalling pathways, and with Aurora-A inhibitors in neuroblastoma [260]. Protein kinases are also frequently observed as off-targets of BETis [261]. In order to exploit the multitargeting capacity of bromodomain inhibitors, several BETis have been investigated as dual inhibitors targeting BRD4 and other proteins in preclinical and clinical trials (reviewed by [262]). Some of the dual inhibitors targeting pBRD4 and PLK1, PI3K, ALK, HDAC, p38α, and CDK9, were reported to have a strong downregulation effect on c-Myc or N-Myc in acute myeloid leukaemia, neuroblastoma, castration-resistant prostate cancer, pancreatic cancer, hepatocellular, and colorectal carcinoma (reviewed by [262]).

As observed for Aurora-A, PROTAC technologies against BRD4 result in a stronger effect compared to BET inhibition; treatment with the MZ1 degrader induced a dose-dependent decrease in neuroblastoma cell viability, more than was observed after JQ1 administration, and reduced the protein levels of both N- and c-Myc [263]. In contrast to this observation, Devaiah and colleagues observed an increase in c-Myc half-life in MZ1-treated neuroblastoma cells, due to the loss of destabilizing phosphorylation on T58, whereas treatment with JQ1 did not affect c-Myc turnover [58]. Moreover, the BET degrader dBET6 induced apoptosis in chronic myeloid leukaemia cells, whose proliferation is regulated by Myc and BRD4, and overcame osteoblast-mediated resistance of leukaemia stem cells against BCR/ABL1 tyrosine kinase inhibitors more than JQ1 [241,264].

## 6. Conclusions

**Figure 8:**
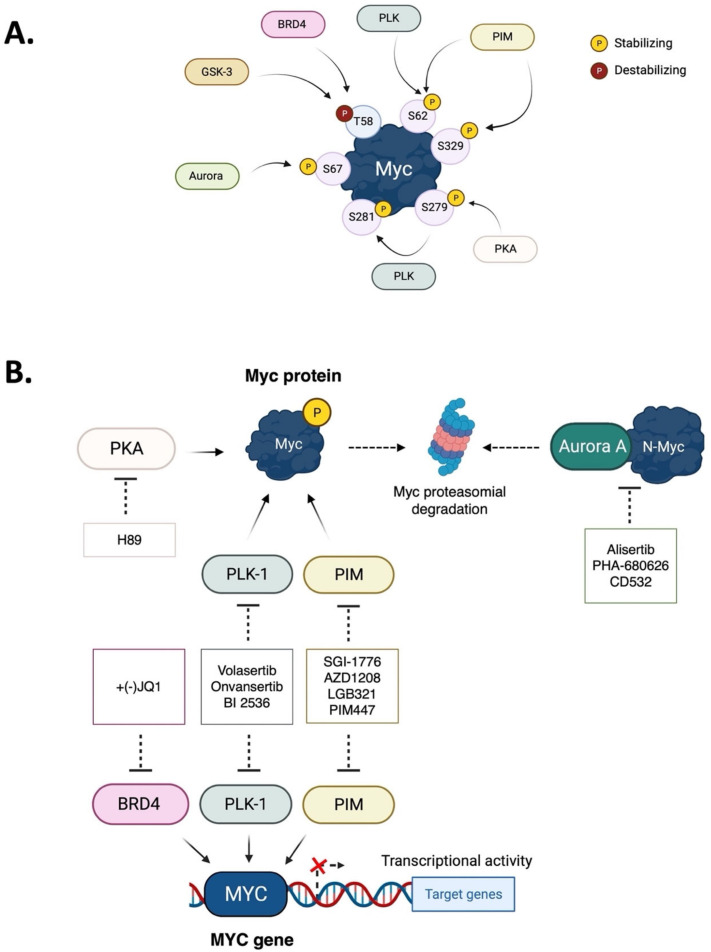
Graphical summary of the kinases and the key phosphorylation sites involved in Myc protein stability are displayed in panel (**A**). Panel (**B**) schematizes the principal inhibitors affecting the pathways exploited to indirectly target Myc at both the protein and gene level. Created with BioRender.com (accessed on 20 January 2023).

Considering their oncogenic properties, the Myc proteins constitute an attractive therapeutic target for cancer treatment (Figure 8). However, the complex and highly dynamic nature of Myc-regulated pathways and genes presents a significant challenge for the development of small molecule inhibitors that can effectively target these proteins. A deeper understanding of the molecular mechanisms by which Myc proteins contribute to oncogenesis is necessary for the development of effective therapeutic strategies. It is clear that Myc protein levels and activity must be tightly regulated in cells, and this regulation is mediated by a variety of molecular signalling pathways. In several types of cancer, high levels of Myc lead to the uncontrolled expression of its target genes, primarily impacting metabolism and proliferation. Interestingly, Myc also upregulates proteins that help to stabilize it, creating a self-perpetuating regulatory loop. These proteins are often protein kinases involved in metabolism and the cell cycle, which are themselves targets for cancer therapy. Therefore, the availability of inhibitors of kinases that influence the stability of Myc family members may provide a promising and feasible indirect approach to target the “undruggable” Myc, with the potential to uncover new therapeutic opportunities for cancer treatment.

## Figures and Tables

**Table 1:** List of kinase inhibitors described to have effects on Myc.

Target Kinase	Inhibitor	Structure	Effects on Myc	Tumour Models	Phase/Status
**PLK1**	BI 2536	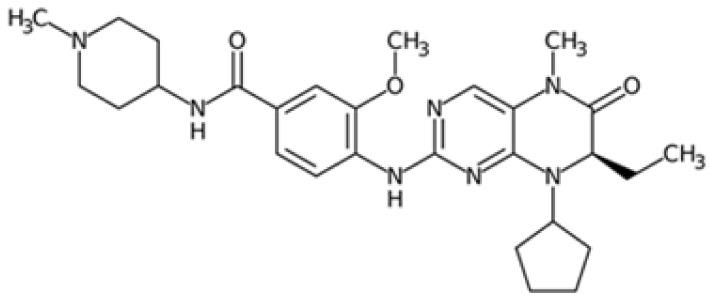	Decreased Myc protein levels, both total protein abundance and phospho-S62Myc; enhanced Myc proteolysis as a result of the stabilization of the ubiquitin ligase Fbxw7; impaired MYC transcriptional activity and downregulation of MYC-target genes	Neuroblastoma [50]	Phase II NCT00376623
	Volasertib (BI 6727)	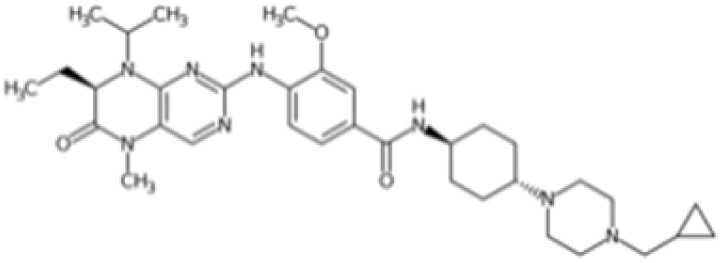		T-lymphoma B-lymphoma Neuroblastoma Rhabdomyosarcoma [50,81,83,206]	Phase I/II NCT01121406 NCT00804856 NCT00824408 NCT00969553
	Onvansertib (PCM-075)	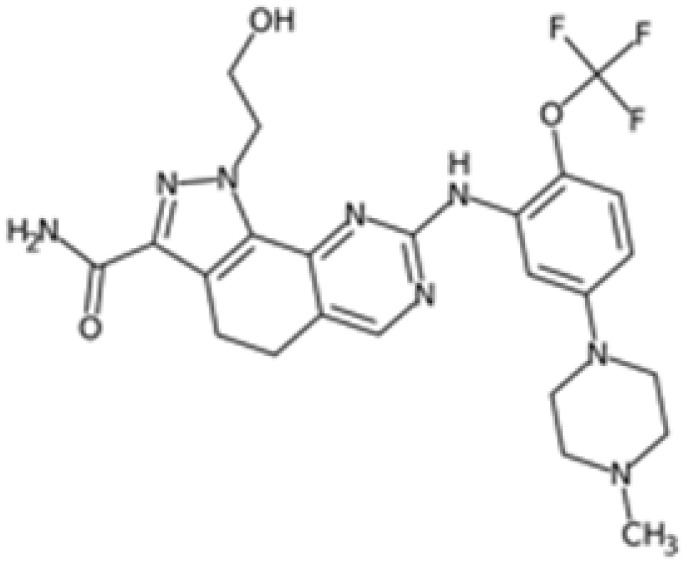		Medulloblastoma [207]	Phase I/II NCT03414034 NCT03829410 NCT04005690 NCT05549661 NCT04752696
**Aurora-A**	Alisertib (MLN8237)	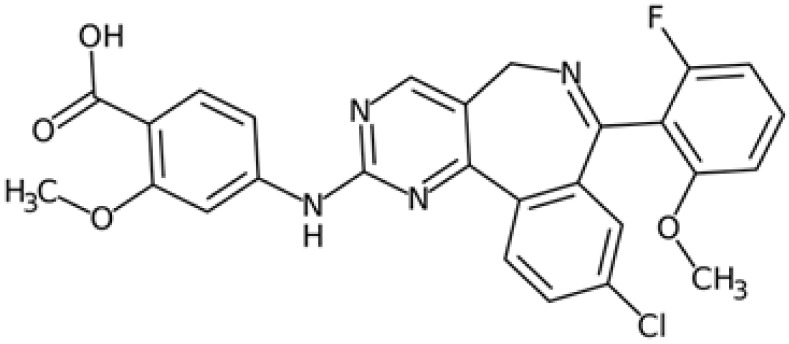	Enhanced degradation of N-Myc protein as a result of the disruption of the complex with Aurora-A, which prevents N-Myc from ubiquitin-proteasome-mediated degradation	Neuroblastoma [123]	Phase I/II/III NCT01799278 NCT01898078 NCT02719691 NCT02293005
	CD532	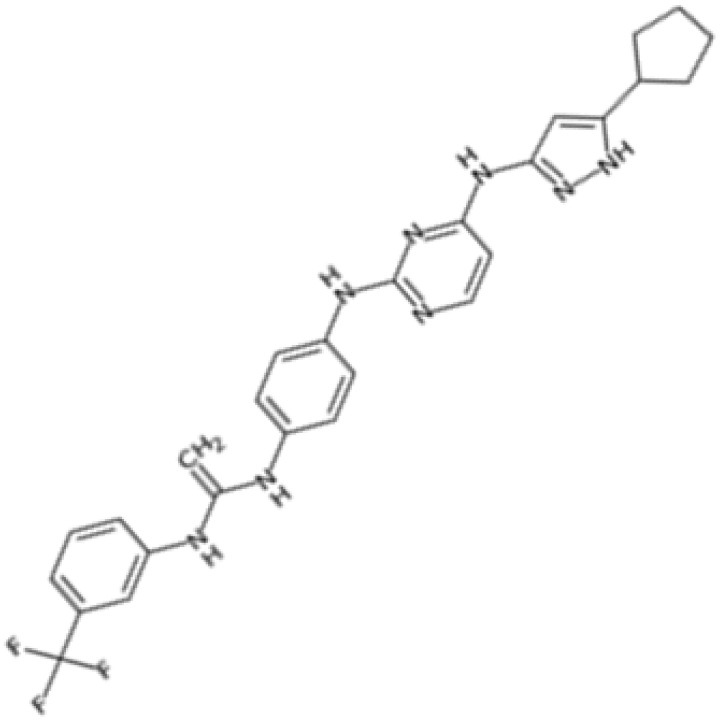		Neuroblastoma Medulloblastoma Neuroendocrine prostate cancer [208,209]	Preclinical
	PHA-680626	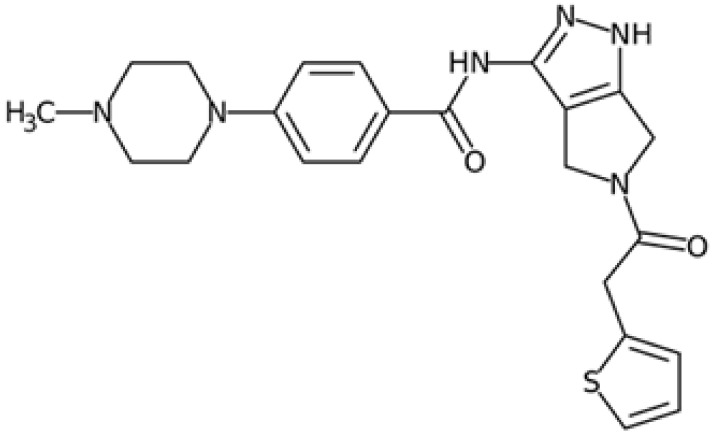		Osteosarcoma Neuroblastoma [210]	Preclinical
**GSK-3**	SB-732881-H	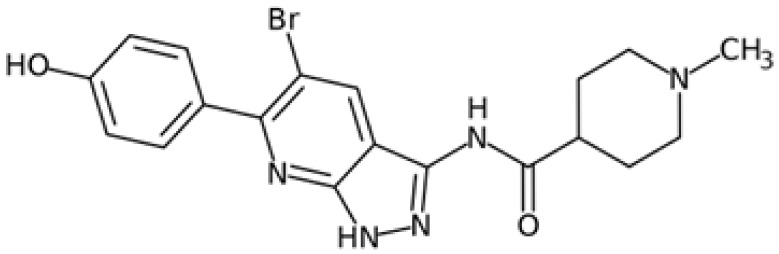	Enhanced cell apoptosis in a c-Myc- and β-catenin-dependent manner	Pancreatic adenocarcinoma Lung cancer [141]	Preclinical
**PKA**	H89	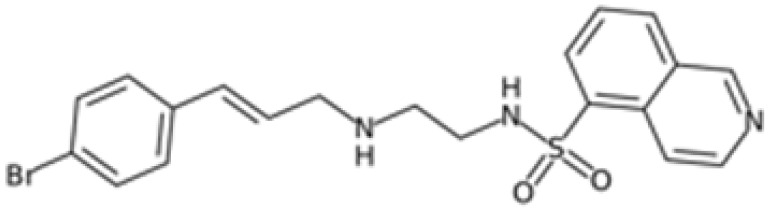	Decreased Myc protein levels as a result of the impaired PKA-mediated stabilization of Myc	Prostate cancer Breast cancer [85]	Preclinical
**PIM**	SGI-1776	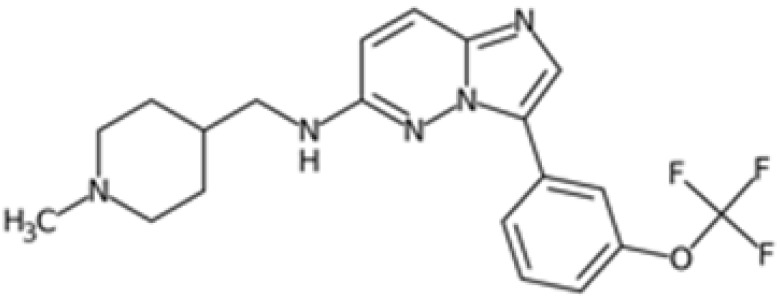	Reduction in total Myc protein abundance as well as phospho-S62Myc; Impaired MYC-driven transcriptional program	Diffuse large B-cell lymphoma Triple-negative breast cancer Chronic lymphocytic leukaemia Mantle cell lymphoma [178,211,212,213]	Phase I—Withdrawn due to cardiac toxicity NCT01239108 NCT00848601
	AZD1208	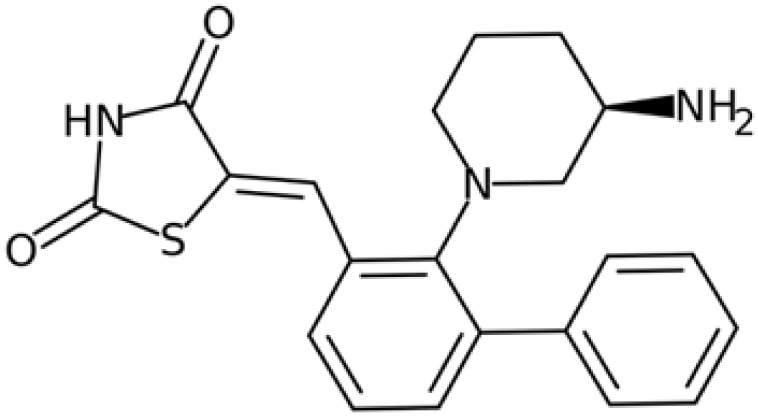		Acute myeloid leukaemia Prostate cancer [214,215]	Phase I NCT01489722 NCT01588548
	LGB321	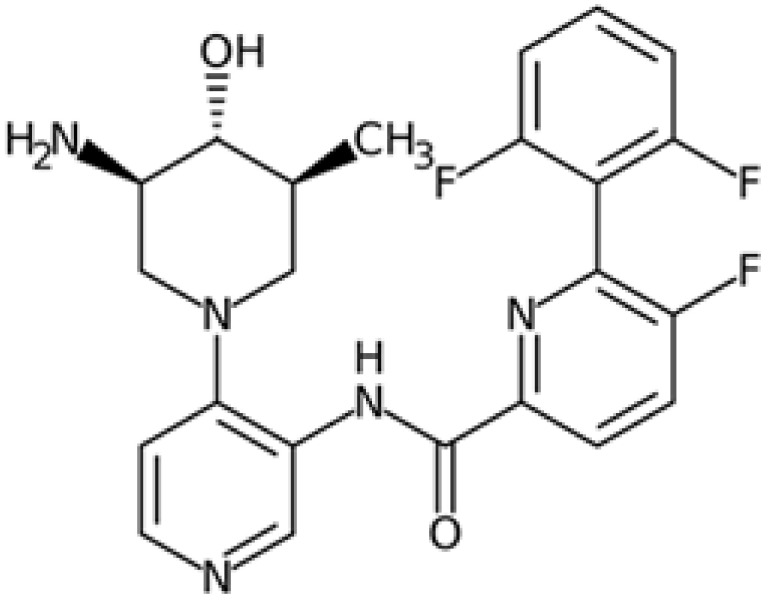		Triple-negative breast cancer [211]	Preclinical
	LGH447 (PIM447)	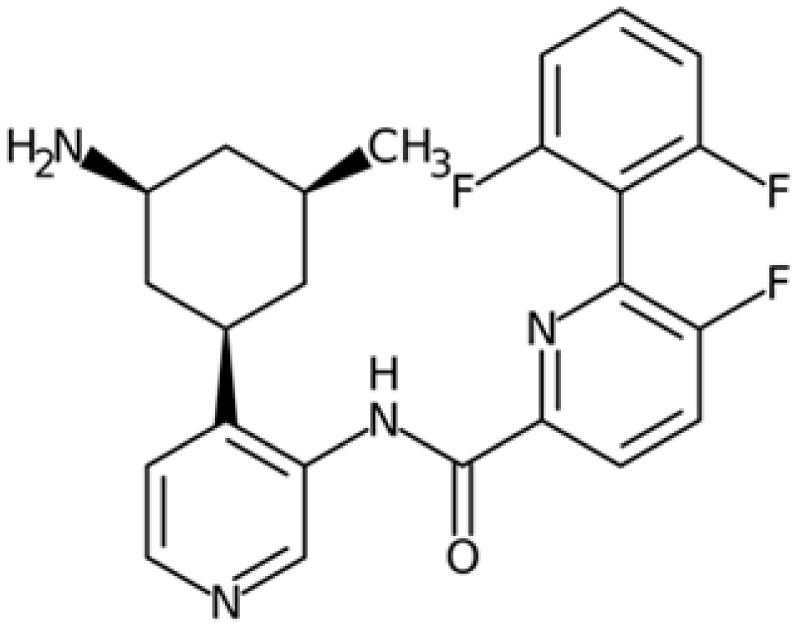		Diffuse large B-cell lymphoma Multiple myeloma [178,216]	Phase I NCT02370706 NCT02160951 NCT02078609 NCT02144038 NCT01456689
	SEL24/ MEN1703	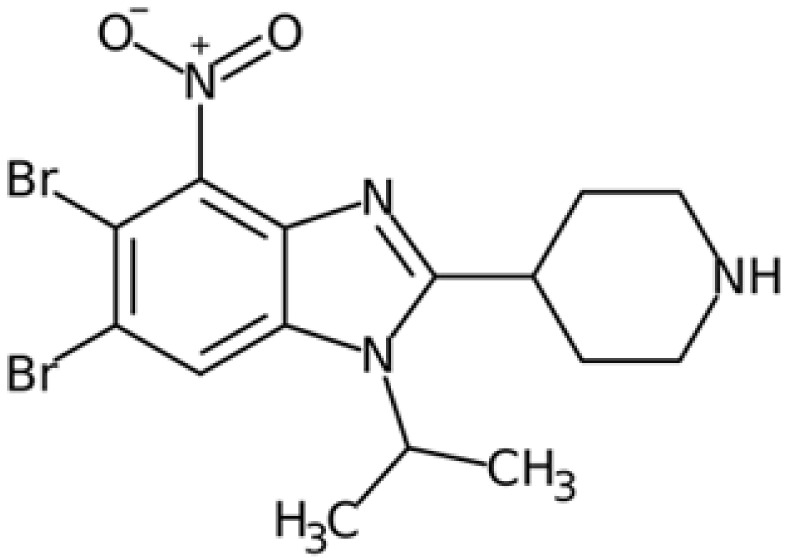	Downregulation of Myc mediated at least in part by increased proteasomal degradation; Impaired MYC-driven transcriptional program	Diffuse large B-cell lymphoma [178]	Phase I/II NCT03008187
**BRD4**	+(-)JQ1	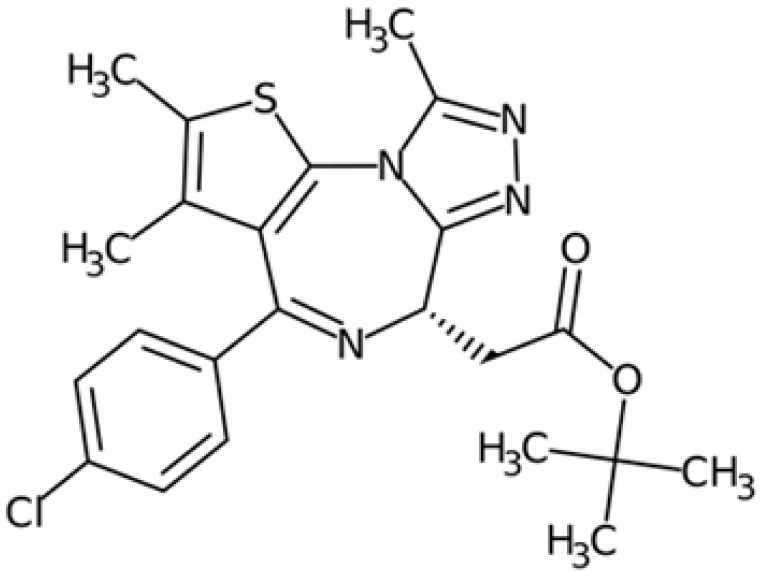	Myc downregulation through BRD4 displacement from MYCN promoter	MYC-amplified neuroblastoma Glioblastoma [217]	Phase I—Derivative inhibitor RO6870810 [218,219] NCT02308761 NCT03068351 NCT01987362 NCT03292172 NCT03255096
**BRD4/** **PLK1**	9b (WNY0824)	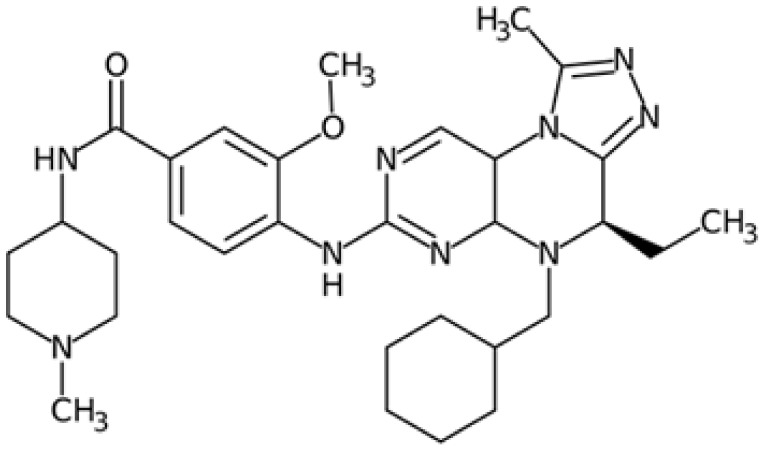	Inhibition of N-Myc and c-Myc transcription	Acute myeloid leukaemia Castration-resistant prostate cancer Colorectal adenocarcinoma Melanoma Ovarian cancer [220,221]	Preclinical
	UMB103	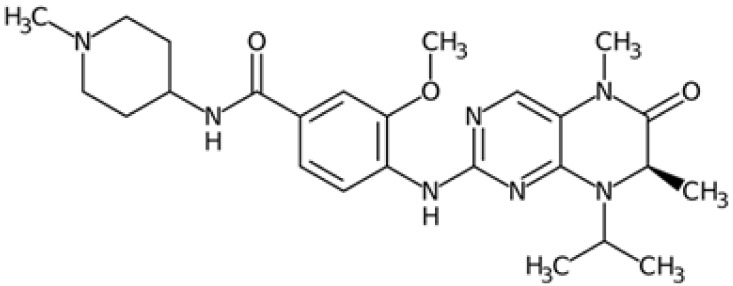	Decreased N-Myc protein levels	Neuroblastoma Rhabdomyosarcoma [206]	Preclinical
	UMB160	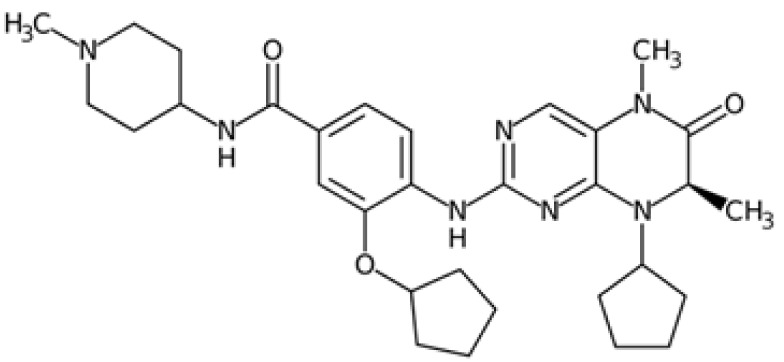			
**BRD4/** **PI3K**	SF2523	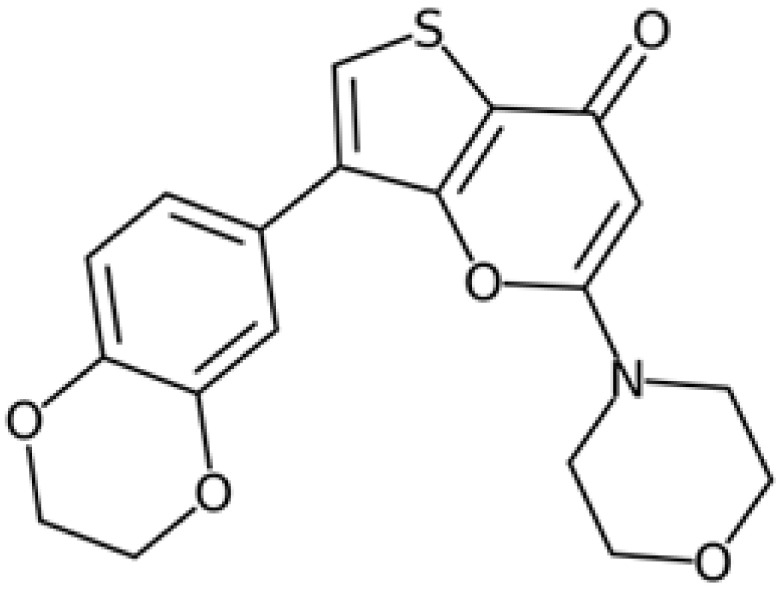	Myc downregulation through BRD4 displacement from MYCN promoter	MYCN-amplified neuroblastoma Pancreatic carcinoma [222]	Preclinical
	SF1126	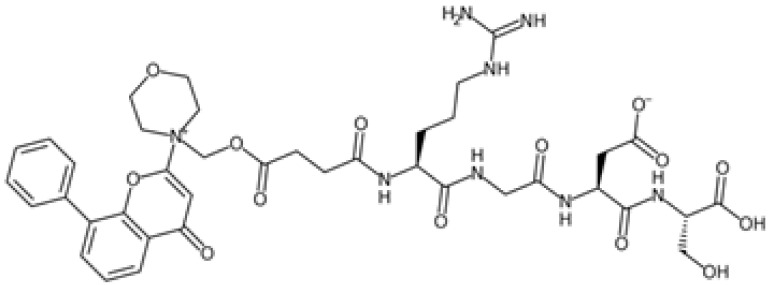		Hepatocellular carcinoma [223]	Phase I/II NCT03059147 NCT02337309 NCT02644122 NCT00907205
**BRD4/** **HDAC**	17c	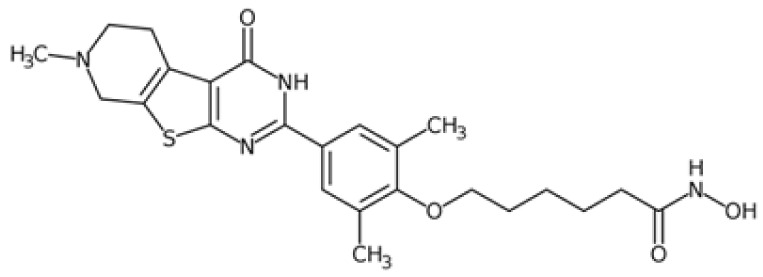	Decrease in c-Myc protein levels	Colorectal cancer [224]	Preclinical
	16ae	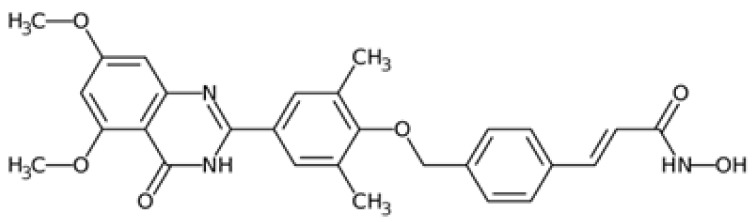	Decrease in Myc protein levels	Acute myeloid leukaemia [225]	Preclinical
	19f	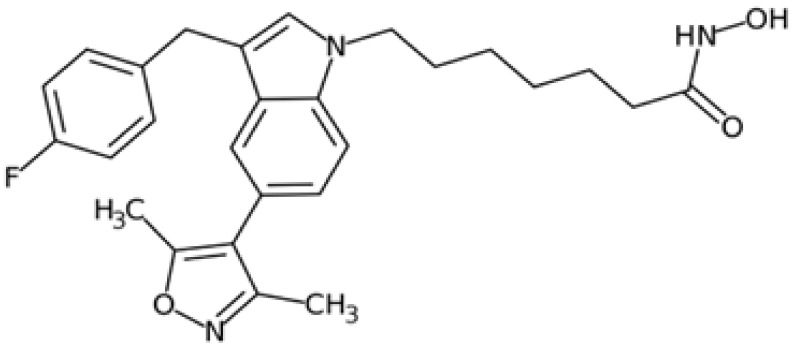	Decrease in c-Myc protein levels	Acute monocytic leukaemia [226]	Preclinical
	13a	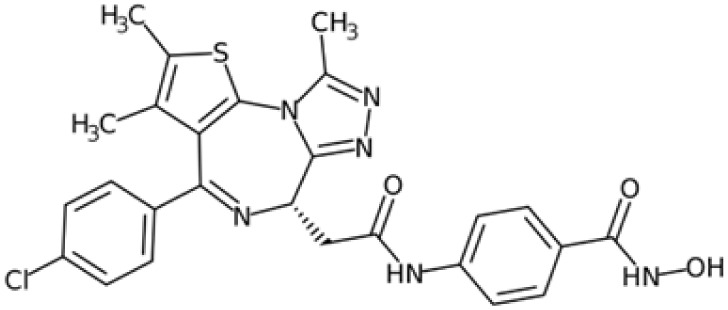	Decrease in c-Myc mRNA and protein levels	Pancreatic cancer [227]	Preclinical
	Compound 40	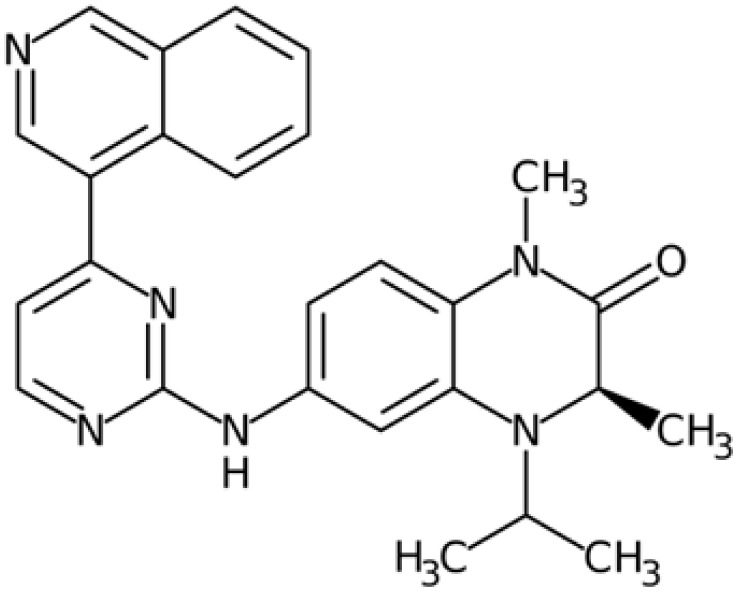	c-Myc downregulation and decrease in mRNA expression	Pancreatic cancer Adenocarcinoma [227]	Preclinical
**BRD4/** **p38α**	SB-284851-BT	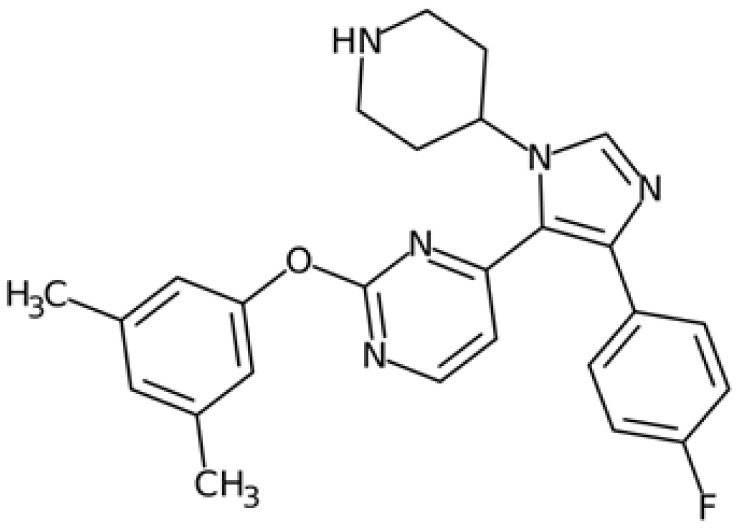	Decrease in c-Myc protein levels	Immunoglobulin A Lambda myeloma [228]	Preclinical

## Data Availability

Not applicable.
